# Supplementary material for: Dibenzocyclooctadiene Lignans from Schisandra chinensis with Anti-Inflammatory Effects
Source: Int J Mol Sci. 2024 Mar 19;25(6):3465. doi: 10.3390/ijms25063465 (PMC10971102; doi:10.3390/ijms25063465)
Supplement: Supplementary file 1 [file ijms-25-03465-s001.zip › ijms-2864807-supplementary.pdf]

Dibenzocyclooctadiene Lignans from *Schisandra chinensis* with Anti-inflammatory Effects

Michal Rybnikář [1,*], Milan Malaník [1], Karel Šmejkal [1], Emil Švajdlenka [1], Polina Shpet [1,2], Pavel Babica[2], Stefano Dall´Acqua [3], Ondřej Smištík [4], Ondřej Jurček [1], and Jakub Treml [4,*]

Supplementary data

List of figures:

Figure S1. ^{1}H NMR spectrum of compound **1** in CD_3OD-*d*$_4$ (at 303 K)
Figure S2. HSQCDEPT NMR spectrum of compound **1** in CD_3OD-*d*$_4$ (at 303 K)
Figure S3. HMBC NMR spectrum of compound **1** in CD_3OD-*d*$_4$
Figure S4. Diagnostic HMBC correlations of compound **1** in CD_3OD-*d*$_4$
Figure S5. COSY NMR spectrum of compound **1** in CD_3OD-*d*$_4$
Figure S6. NOESY NMR spectrum of compound **1** in CD_3OD-*d*$_4$
Figure S7. HPLC-ELSD chromatogram of compound **1**
Figure S8. UV spectrum of compound **1**
Figure S9. LC-MS and HRMS spectrum of compound **1**
Figure S10. ^{1}H NMR spectrum of compound **2** in $CDCl_3$-*d*
Figure S11. HMBC NMR spectrum of compound **2** in $CDCl_3$-*d*
Figure S12. HQSC NMR spectrum of compound **2** in $CDCl_3$-*d*
Figure S13. COSY NMR spectrum of compound **2** in $CDCl_3$-*d*
Figure S14. NOESY NMR spectrum of compound **2** in $CDCl_3$-*d*
Figure S15. HPLC-DAD chromatogram of compound **2**
Figure S16. LR-MS spectrum of compound **2**
Figure S17. CD spectrum of compound **2**
Figure S18. IR spectrum of compound **2**
Figure S19. ^{1}H NMR spectrum of compound **3** in $CDCl_3$-*d*
Figure S20. HMBC NMR spectrum of compound **3** in $CDCl_3$-*d*
Figure S21. HSQC NMR spectrum of compound **3** in $CDCl_3$-*d*
Figure S22. COSY NMR spectrum of compound **3** in $CDCl_3$-*d*
Figure S23. NOESY NMR spectrum of compound **3** in $CDCl_3$-*d*
Figure S24. HPLC-DAD chromatogram of compound **3**
Figure S25. LR-MS spectrum of compound **3**
Figure S26. CD spectrum of compound **3**
Figure S27. IR spectrum of compound **3**
Figure S28. ^{1}H NMR spectrum of compound **4** in CD_3OD-*d*$_4$
Figure S29. HMBC NMR spectrum of compound **4** in CD_3OD-*d*$_4$
Figure S30. HSQC NMR spectrum of compound **4** in CD_3OD-*d*$_4$
Figure S31. COSY NMR spectrum of compound **4** in CD_3OD-*d*$_4$
Figure S32. NOESY NMR spectrum of compound **4** in CD_3OD-*d*$_4$
Figure S33. HPLC-DAD chromatogram of compound **4**
Figure S34. LR-MS chromatogram of compound **4**
Figure S35. CD spectrum of compound **4**
Figure S36. IR spectrum of compound **4**
Figure S37. ^{1}H NMR spectrum of compound **5** in CD_3OD-*d*$_4$
Figure S38. HMBC NMR spectrum of compound **5** in CD_3OD-*d*$_4$
Figure S39. HSQC NMR spectrum of compound **5** in CD_3OD-*d*$_4$
Figure S40. COSY NMR spectrum of compound **5** in CD_3OD-*d*$_4$
Figure S41. NOESY NMR spectrum of compound **5** in CD_3OD-*d*$_4$
Figure S42. HPLC-DAD chromatogram of compound **5**
Figure S43. LR-MS spectrum of compound **5**
Figure S44. CD spectrum of compound **5**
Figure S45. IR spectrum of compound **5**

Figure S46. HPLC-DAD chromatogram of common injection of compound **6** with standard of (+)-deoxyschisandrin; UV spectrum of peak corresponding to (+)-deoxyschisandrin.
Figure S47. HPLC-DAD chromatogram of common injection of (−)-wuweizisu C with standard of (−)-wuweizisu C; UV spectrum of peak corresponding to (−)-wuweizisu C.
Figure S48. ^{1}H NMR spectrum of epigomisin O in $CDCl_3$-*d*
Figure S49. HMBC NMR spectrum of epigomisin O in $CDCl_3$-*d*
Figure S50. HSQC NMR spectrum of epigomisin O in $CDCl_3$-*d*
Figure S51. COSY NMR spectrum of epigomisin O in $CDCl_3$-*d*
Figure S52. NOESY NMR spectrum of epigomisin O in $CDCl_3$-*d*
Figure S53. HPLC-DAD chromatogram of epigomisin O
Figure S54. LR-MS spectrum of epigomisin O
Figure S55. CD spectrum of epigomisin O
Figure S56. IR spectrum of epigomisin O
Figure S57. ^{1}H NMR spectrum of arisantetralone C in CD Cl_3-*d*
Figure S58. HMBC NMR spectrum of arisantetralone C in $CDCl_3$-*d*
Figure S59. HSQC NMR spectrum of arisantetralone C in $CDCl_3$-*d*
Figure S60. COSY NMR spectrum of arisantetralone C in $CDCl_3$-*d*
Figure S62. NOESY NMR spectrum of arisantetralone C in $CDCl_3$-*d*
Figure S63. HPLC-DAD chromatogram of arisantetralone C
Figure S64. LR-MS spectrum of arisantetralone C
Figure S65. CD spectrum of arisantetralone C
Figure S66. IR spectrum of arisantetralone C
Figure S67. ^{1}H NMR spectrum of arisantetralone A in DMSO-d_6
Figure S68. HMBC NMR spectrum of arisantetralone A in DMSO-d_6
Figure S69. HSQC NMR spectrum of arisantetralone A in DMSO-d_6
Figure S70. COSY NMR spectrum of arisantetralone A in DMSO-d_6
Figure S71. NOESY NMR spectrum of arisantetralone A in DMSO-d_6
Figure S72. HPLC-DAD chromatogram of arisantetralone A
Figure S73. LR-MS spectrum of arisantetralone A
Figure S74. CD spectrum of arisantetralone A
Figure S75. IR spectrum of arisantetralone A
Figure S76. ^{1}H NMR spectrum of (−)-schisantherin E in DMSO-d_6
Figure S77. HMBC NMR spectrum of (−)-schisantherin E in DMSO-d_6
Figure S78. HSQC NMR spectrum of (−)-schisantherin E in DMSO-d_6
Figure S79. COSY NMR spectrum of (−)-schisantherin E in DMSO-d_6
Figure S80. HPLC-DAD chromatogram of (−)-schisantherin E
Figure S81. LR-MS spectrum of (−)-schisantherin E
Figure S82. CD spectrum of (−)-schisantherin E
Figure S83. IR spectrum of (−)-schisantherin E
Figure S84. Schema of isolation and total yields of compounds **2–6**, (−)-wuweizisu C, arisantetralone A, arisantetralone C, epigomisin O, and (−)-schisantherin E
Figure S85. Schema of isolation and yield of compound **1**
Figure S86. Antiproliferative activity of lignans **2–14**
Table S1. Chromatographic conditions of separation of compounds **2, 6** and arisantetralone C from fraction SC 122-123
Table S2. Chromatographic conditions of separation of compounds **3, 4, 5** and **6** from fraction SC 130
Table S3. Chromatographic conditions of purification of compound **3** from fraction SC 130
Table S4. Chromatographic conditions of the first purification of compounds **4** and **5** from fraction SC 130
Table S5. Chromatographic conditions of the second purification of compounds **4** and **5** from fraction SC 130
Table S6. Chromatographic conditions of separation of compound **6** and (−)-wuweizisu C from fraction SC 120-121
Table S7. Chromatographic conditions of separation of epigomisin O from fraction SC 138-148
Table S8. Chromatographic conditions of separation of arisantetralone A from fraction SC 149-167
Table S9 Chromatographic conditions of separation of (−)-schisantherin E from fraction SC 175-181
Table S10. Chromatographic conditions of separation of compounds **3, 4, 5** and **6** from fraction SC 126-129
Table S11. Chromatographic conditions of purification of compounds **4** and **5** from fraction SC 126-129

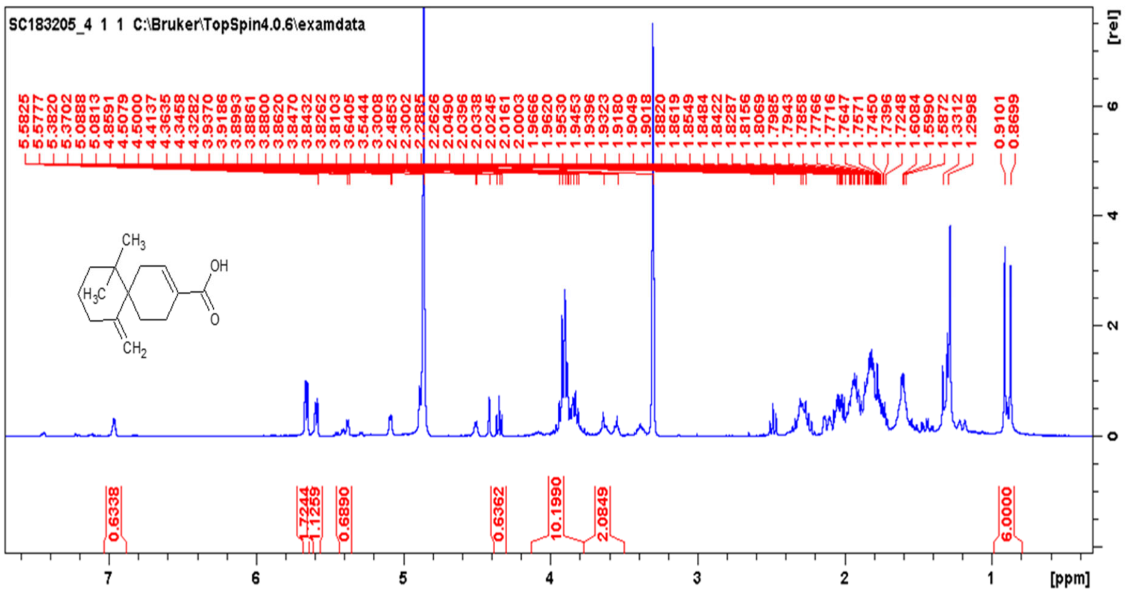

Figure S1. ^{1}H NMR spectrum of compound **1** in CD_3OD (at 303 K)

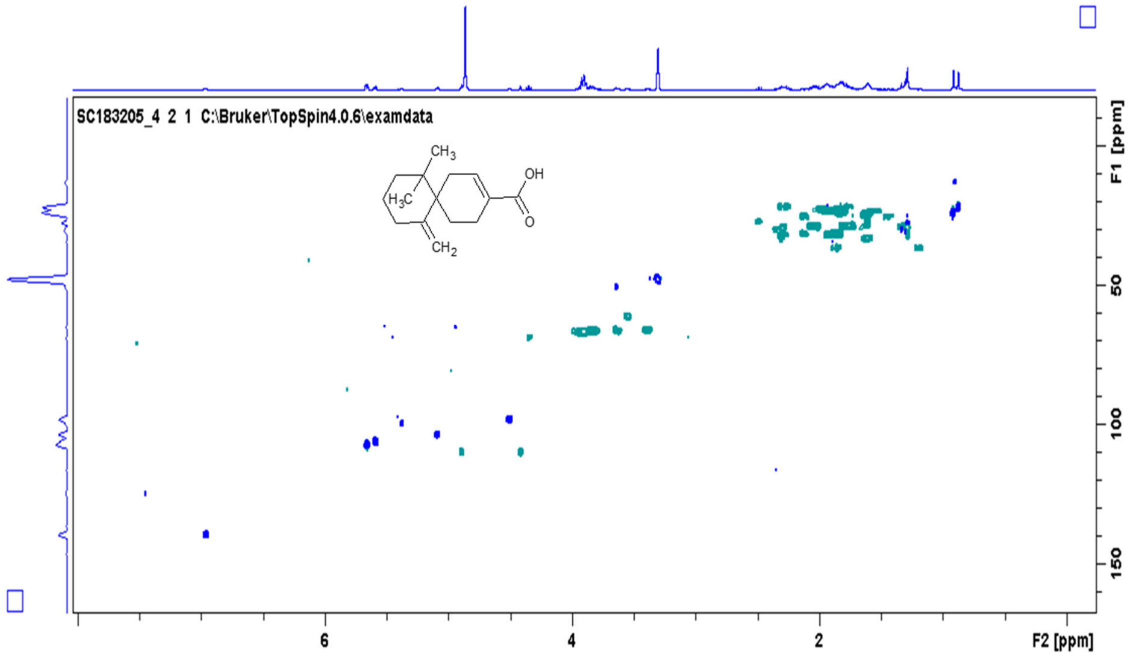

Figure S2. HSQCDEPT NMR spectrum of compound **1** in CD_3OD (at 303 K)

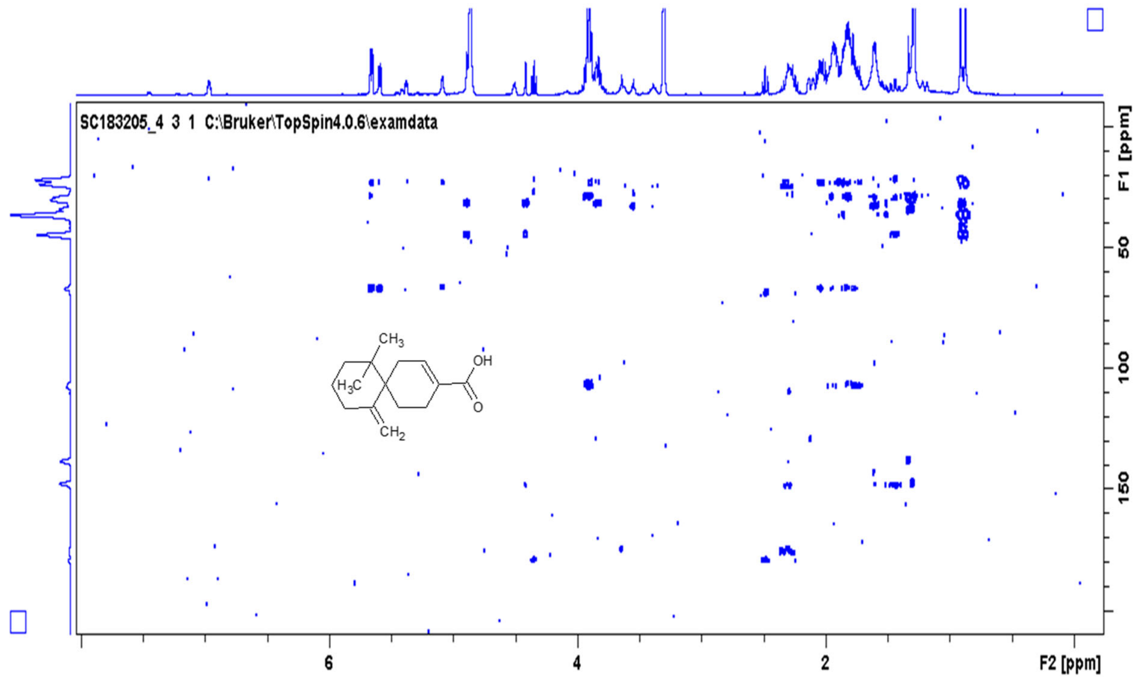

Figure S3. HMBC NMR spectrum of compound **1** in CD_3OD

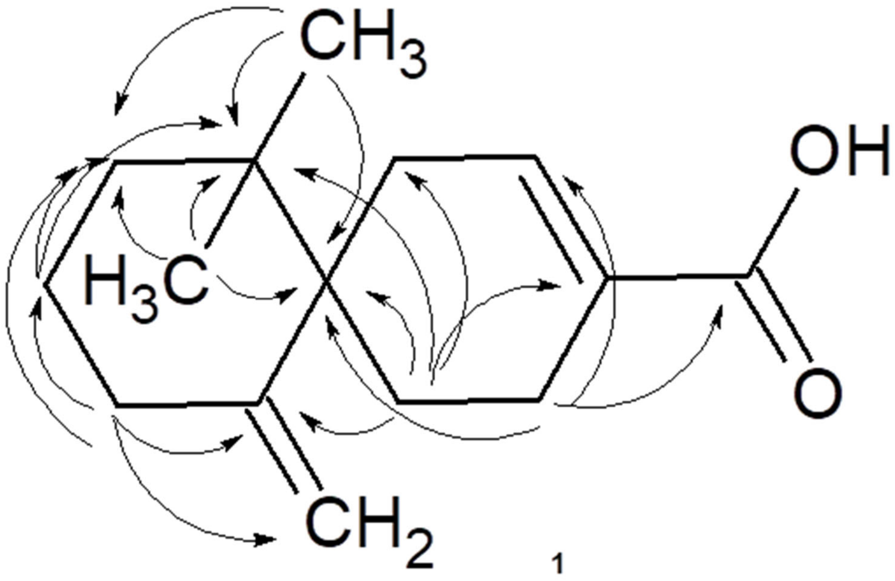

Figure S4. Diagnostic HMBC correlations of compound **1** in CD_3OD

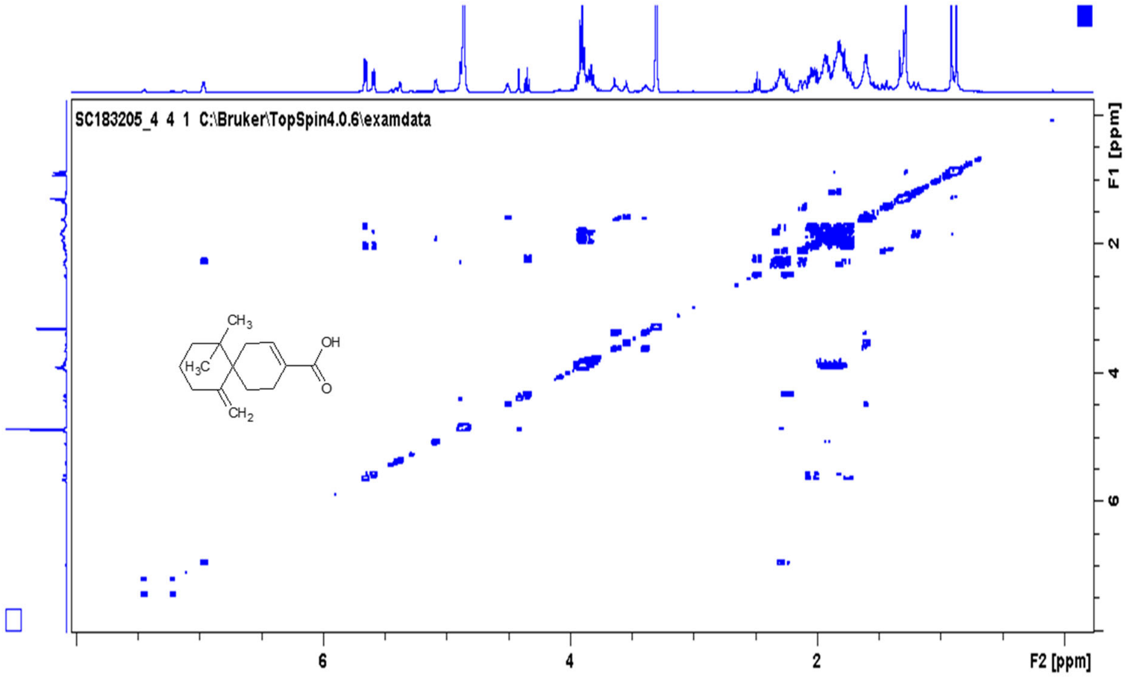

Figure S5. COSY NMR spectrum of compound **1** in CD_3OD

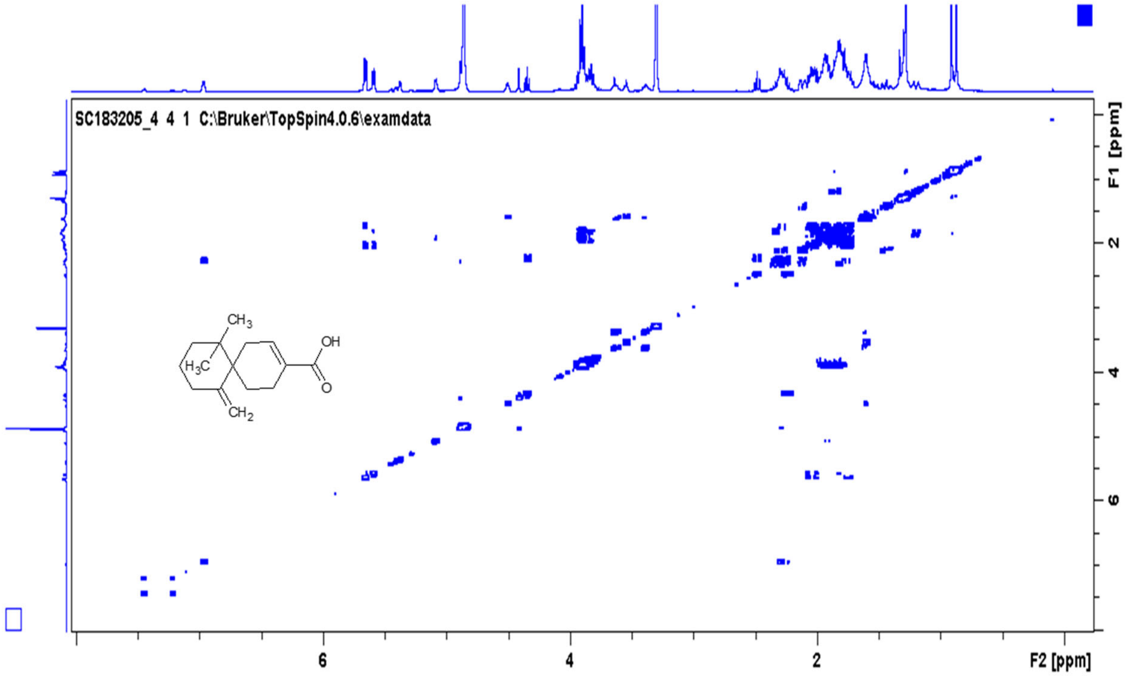

Figure S6. NOESY NMR spectrum of compound **1** in CD_3OD

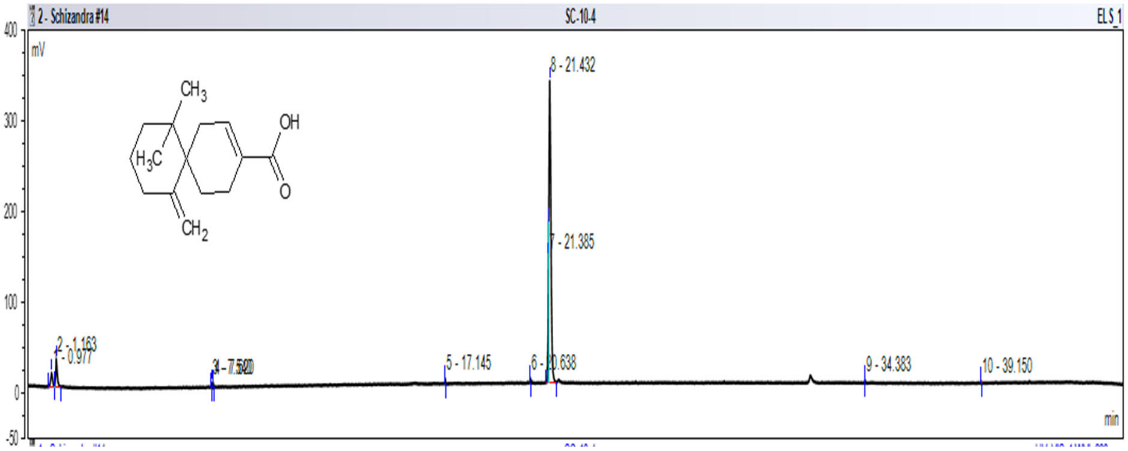

Figure S7. HPLC-ELSD chromatogram of compound **1**

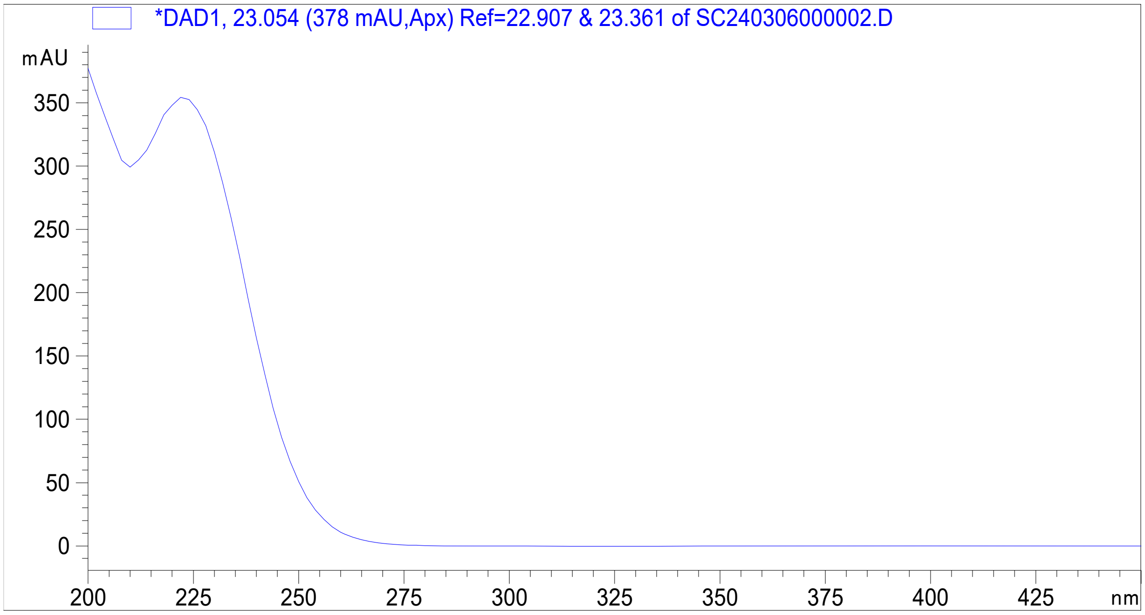

Figure S8. UV spectrum of compound **1**

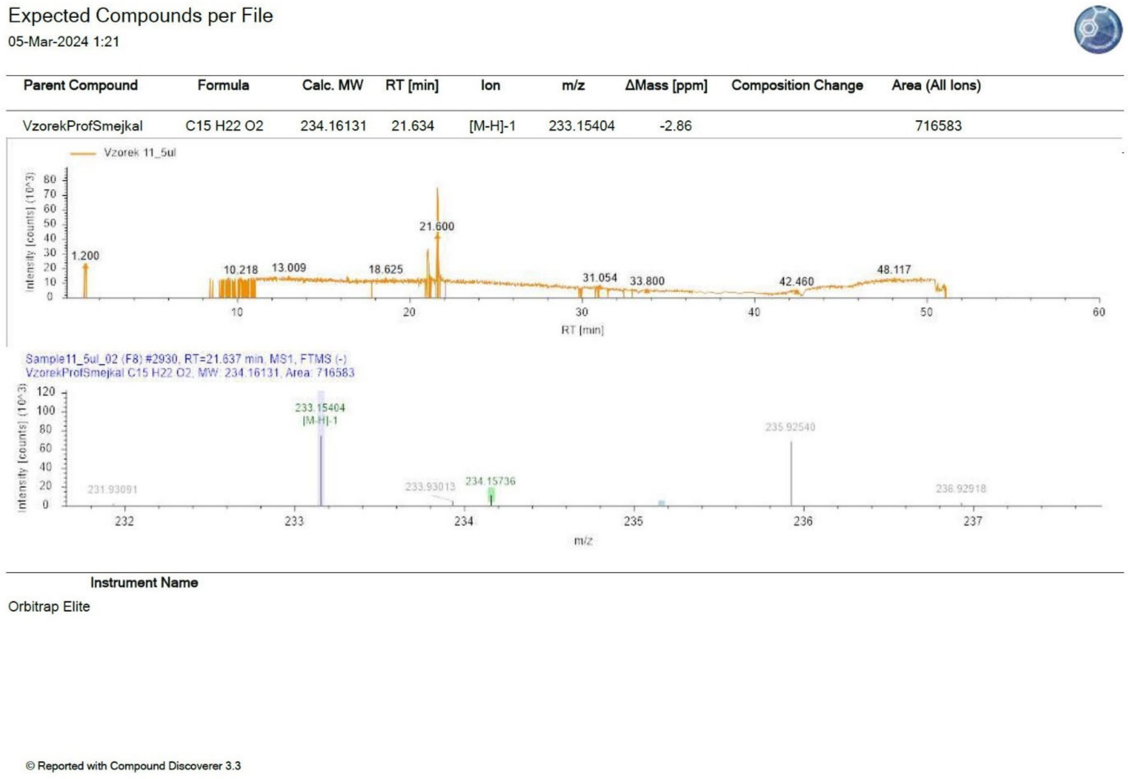

Figure S9. LC-MS and HRMS spectrum of compound **1**

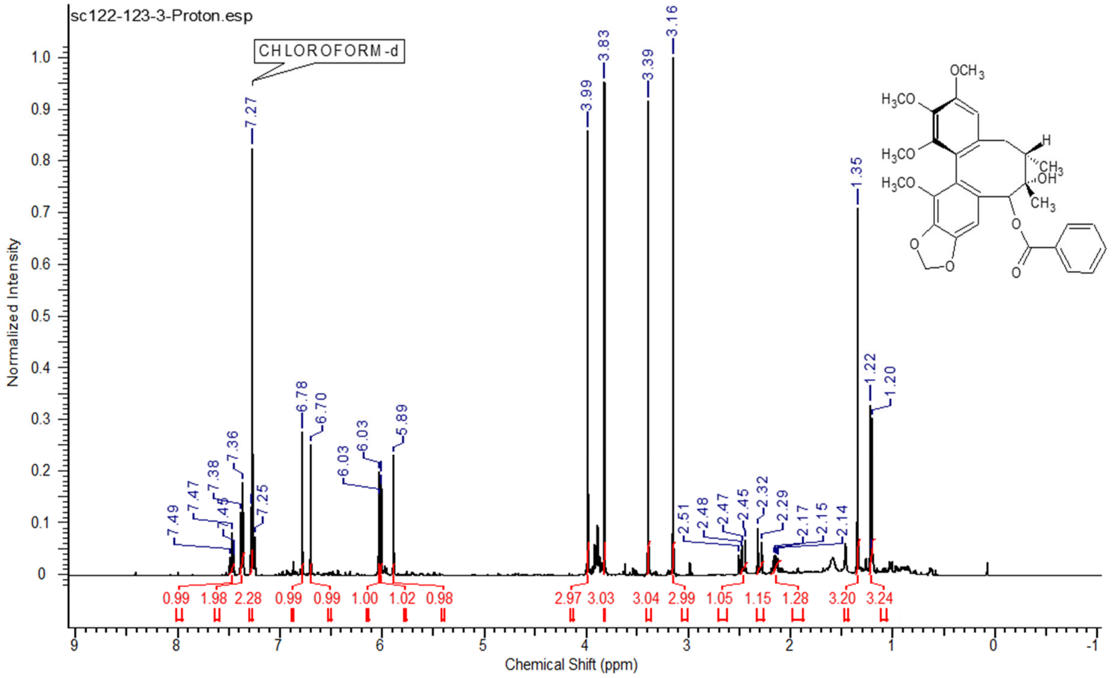

Figure S10. ^{1}H NMR spectrum of compound **2** in $CDCl_3$

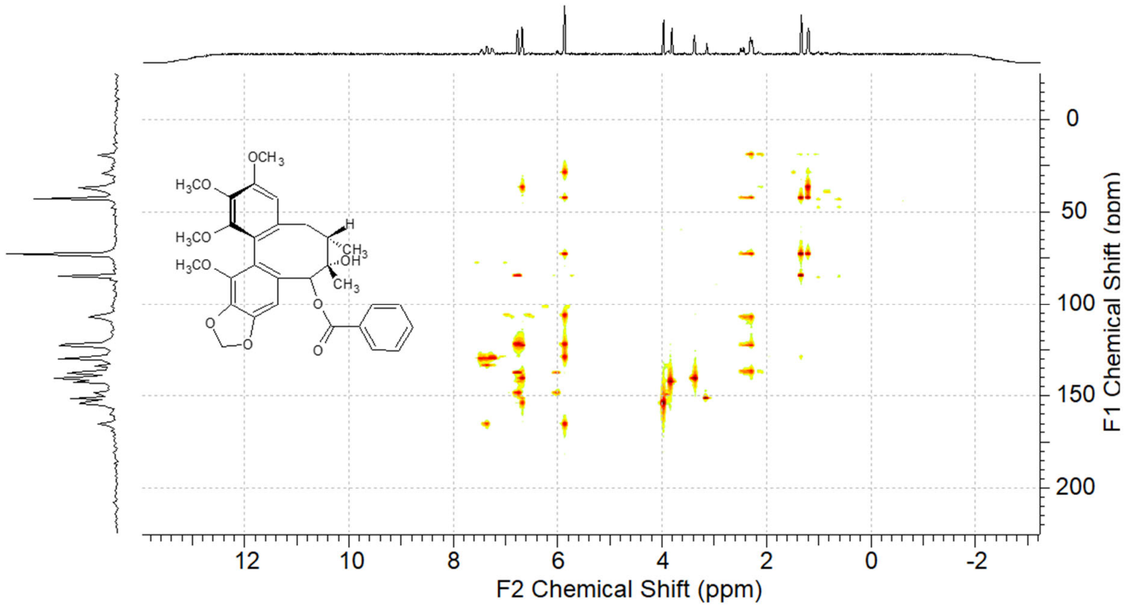

Figure S11. HMBC NMR spectrum of compound **2** in $CDCl_3$

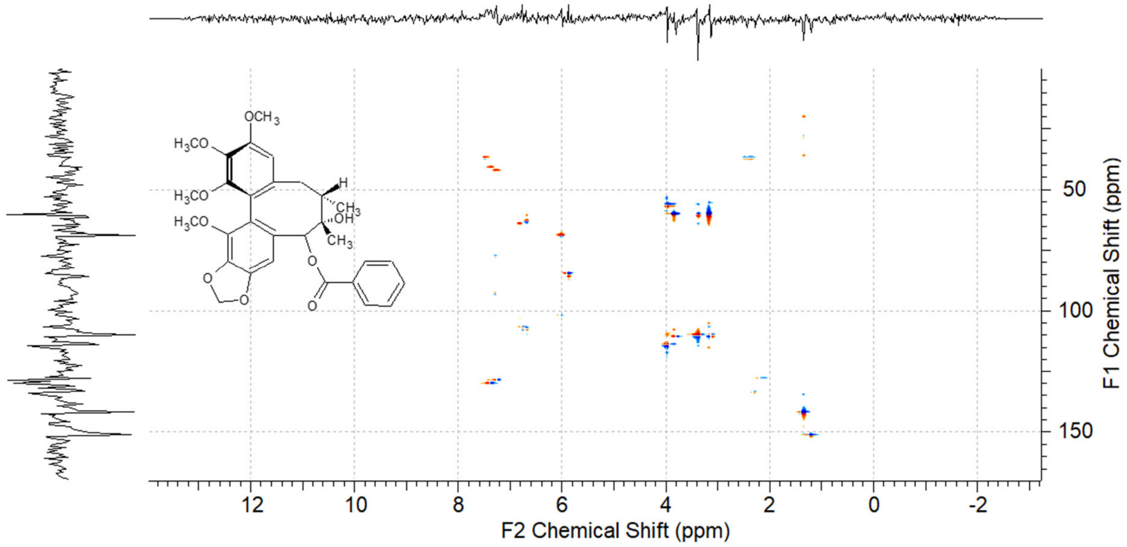

Figure S12. HQSC NMR spectrum of compound **2** in $CDCl_3$

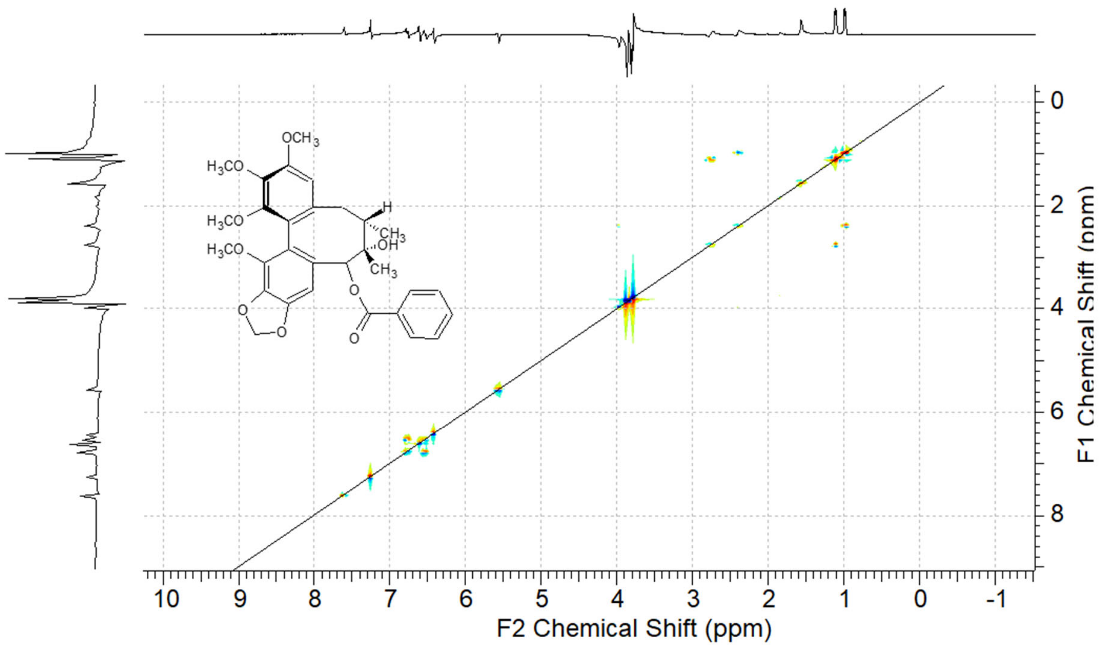

Figure S13. COSY NMR spectrum of compound **2** in $CDCl_3$

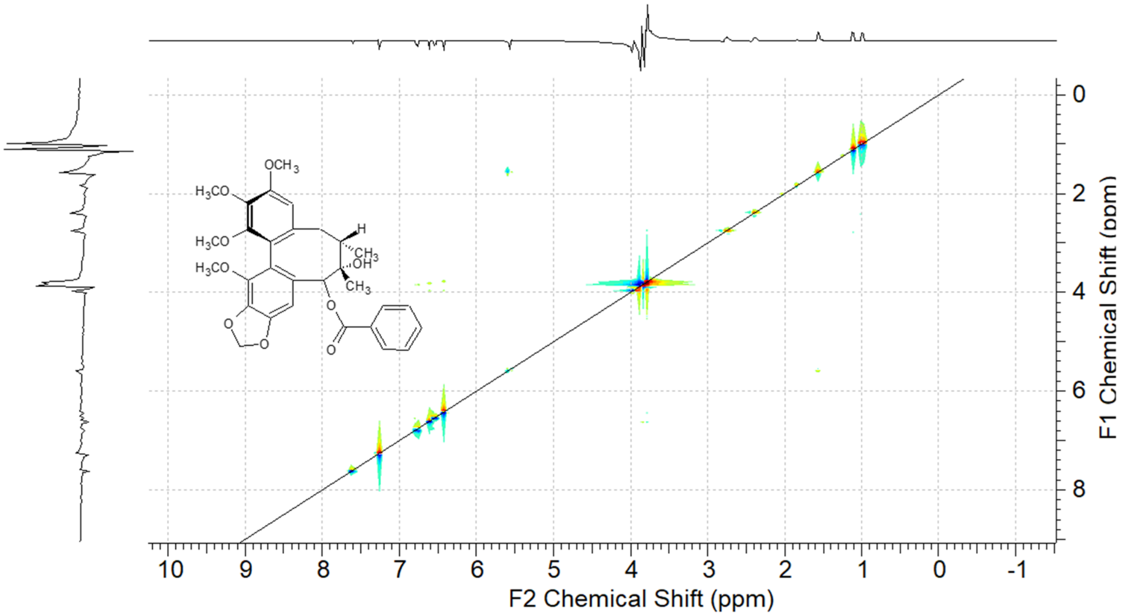

Figure S14. NOESY NMR spectrum of compound **2** in $CDCl_3$

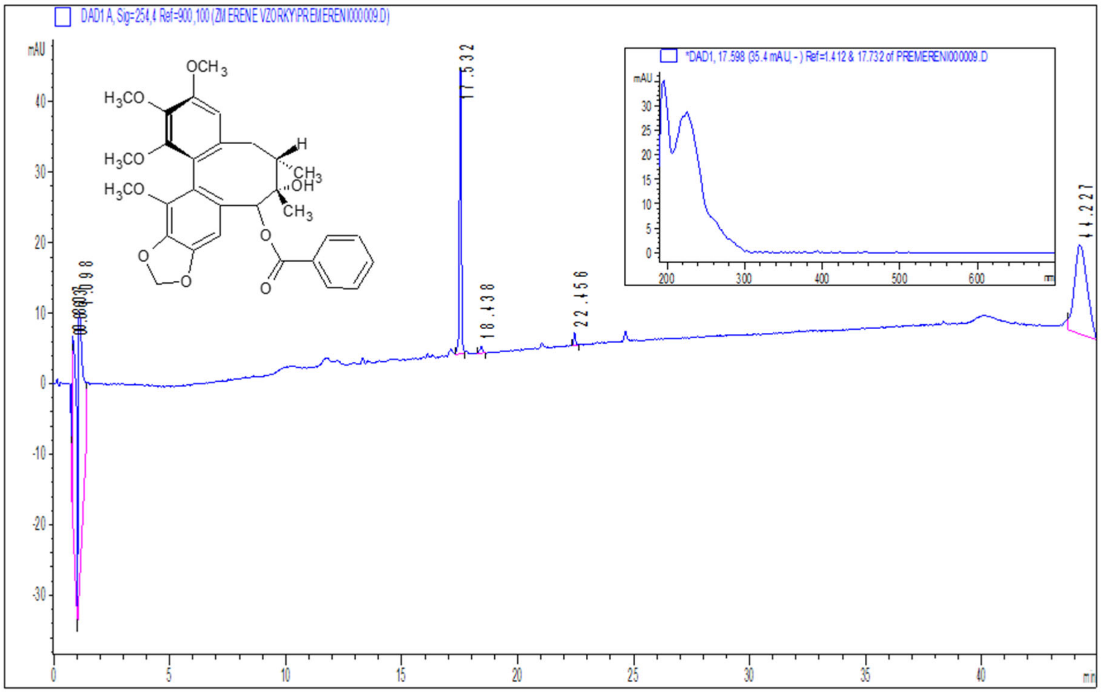

Figure S15. HPLC-DAD chromatogram of compound **2**

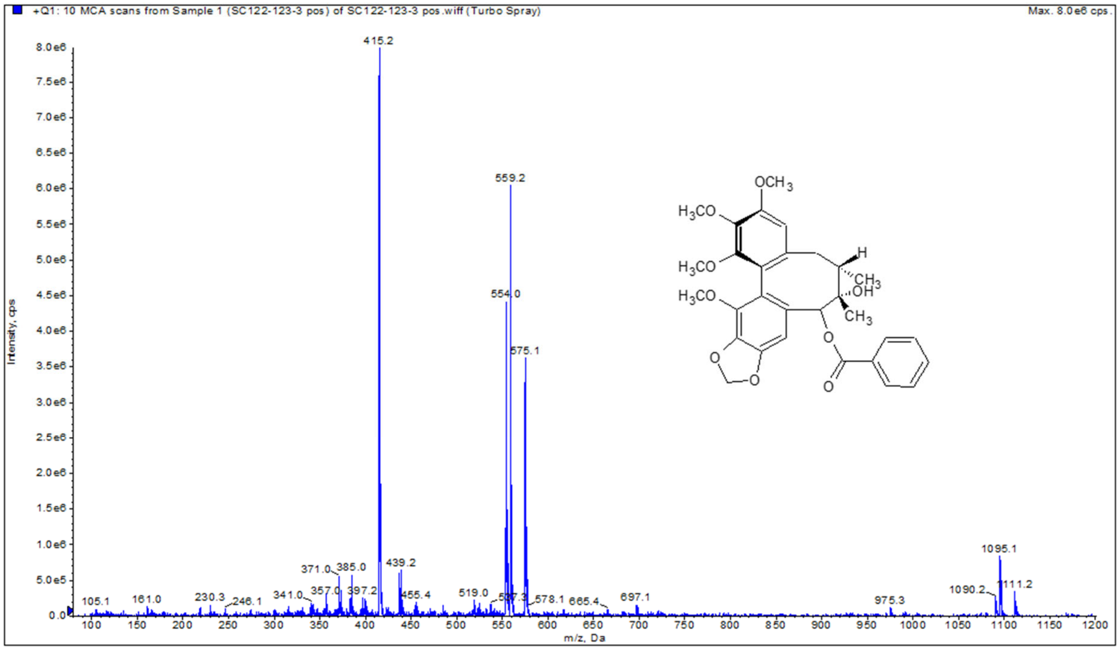

Figure S16. LR-MS spectrum of compound **2**

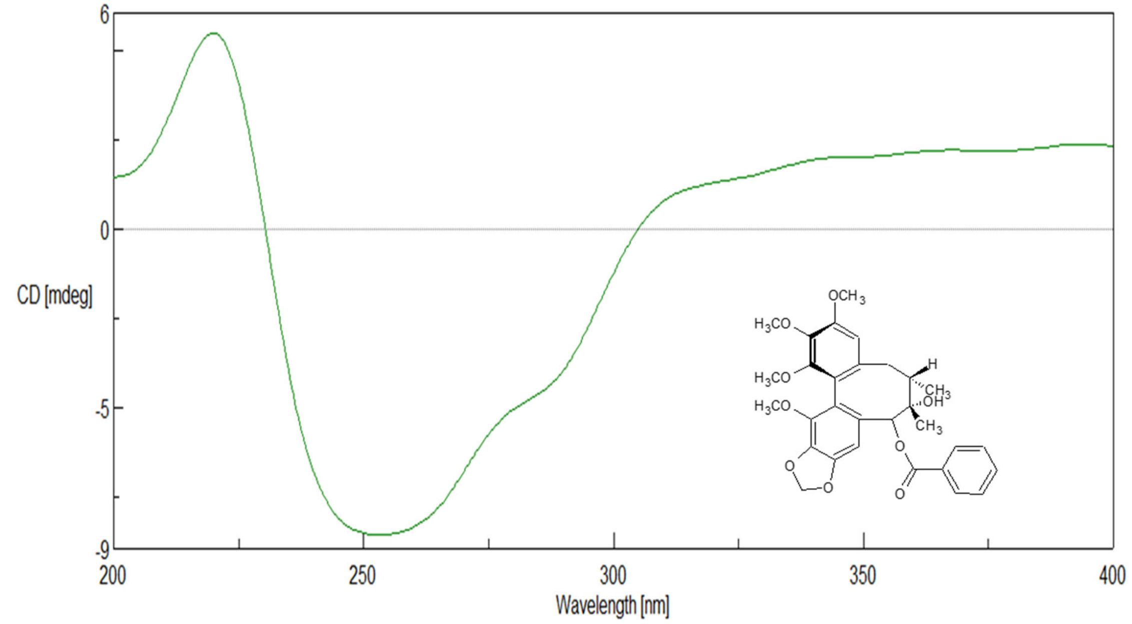

Figure S17. CD spectrum of compound **2** in methanol (c= 1 mg/mL)

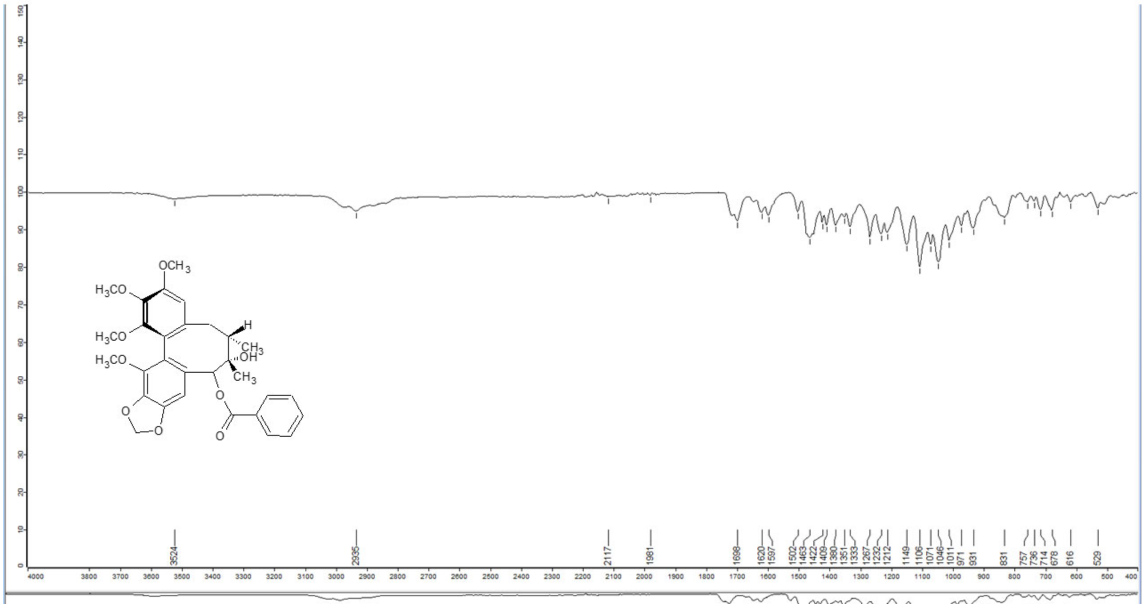

Figure S18. IR spectrum of compound **2**

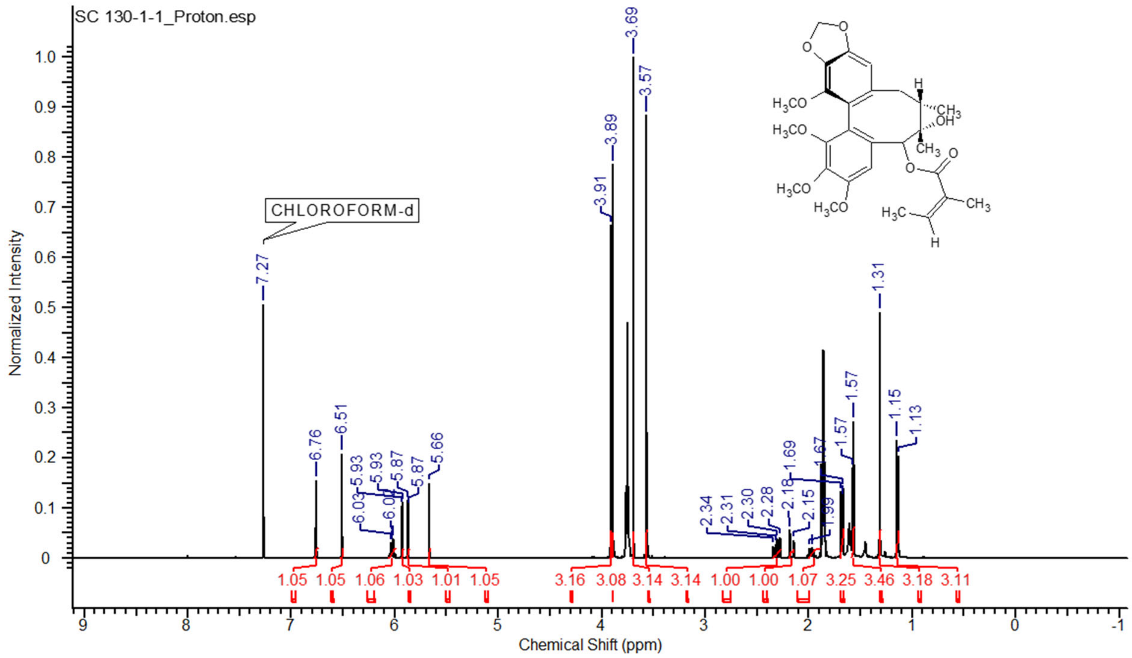

Figure S19. ^{1}H NMR spectrum of compound **3** in $CDCl_3$

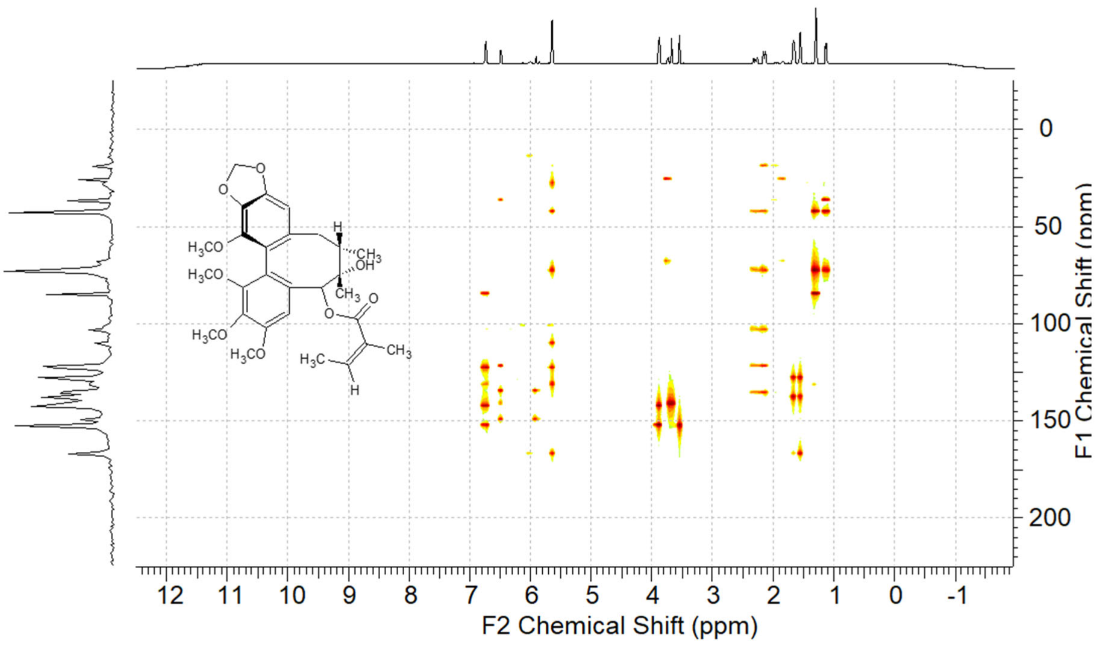

Figure S20. HMBC NMR spectrum of compound **3** in $CDCl_3$

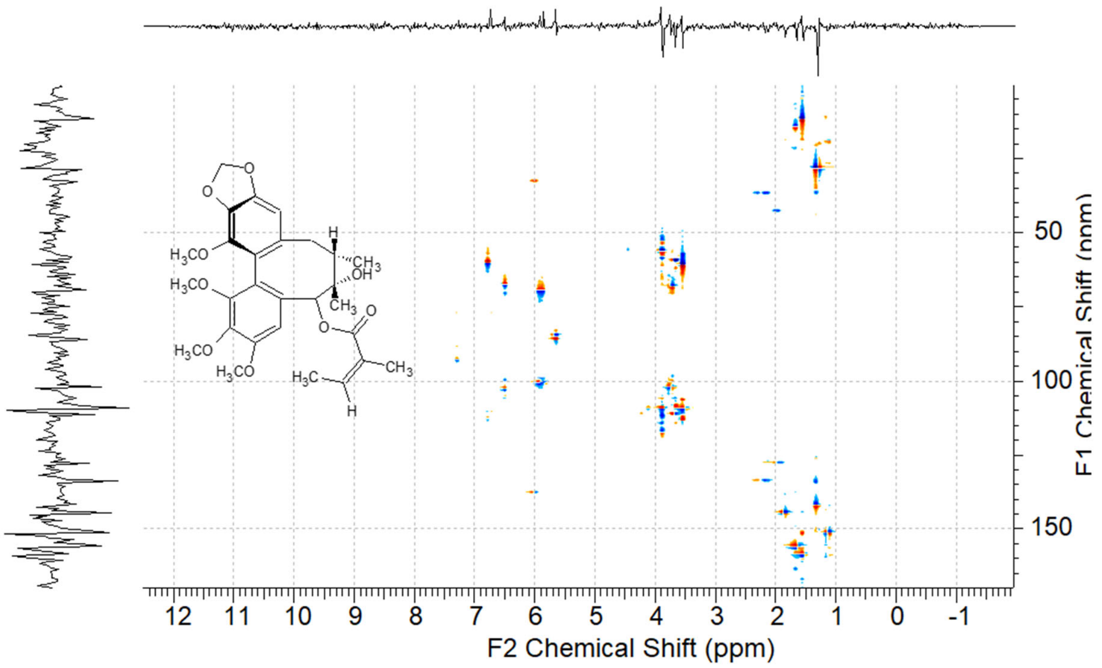

Figure S21. HSQC NMR spectrum of compound **3** in $CDCl_3$

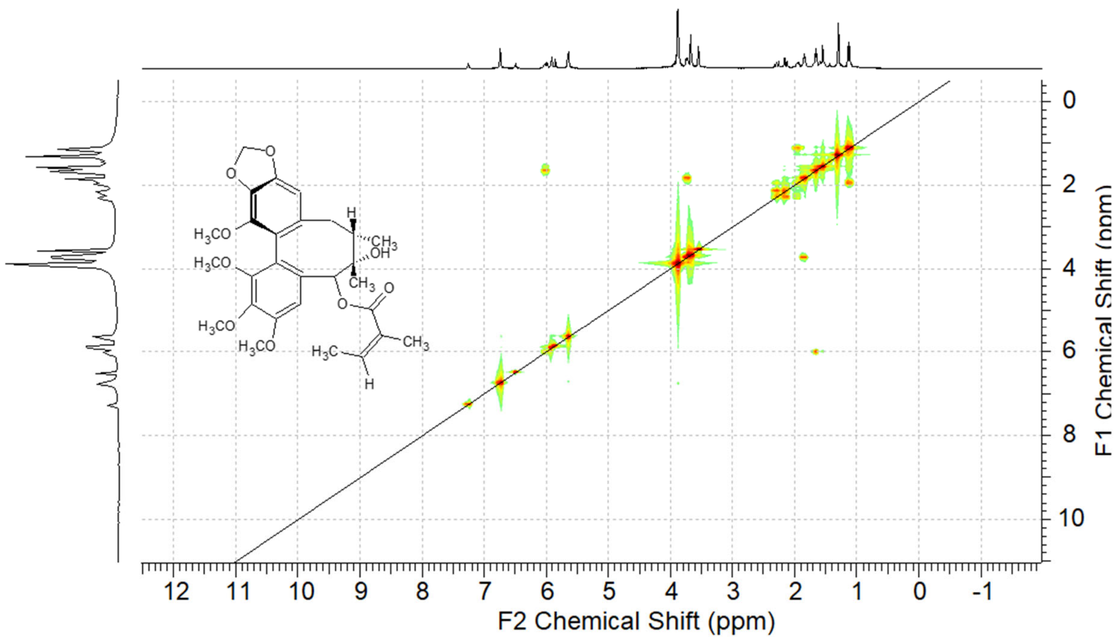

Figure S22. COSY NMR spectrum of compound **3** in $CDCl_3$

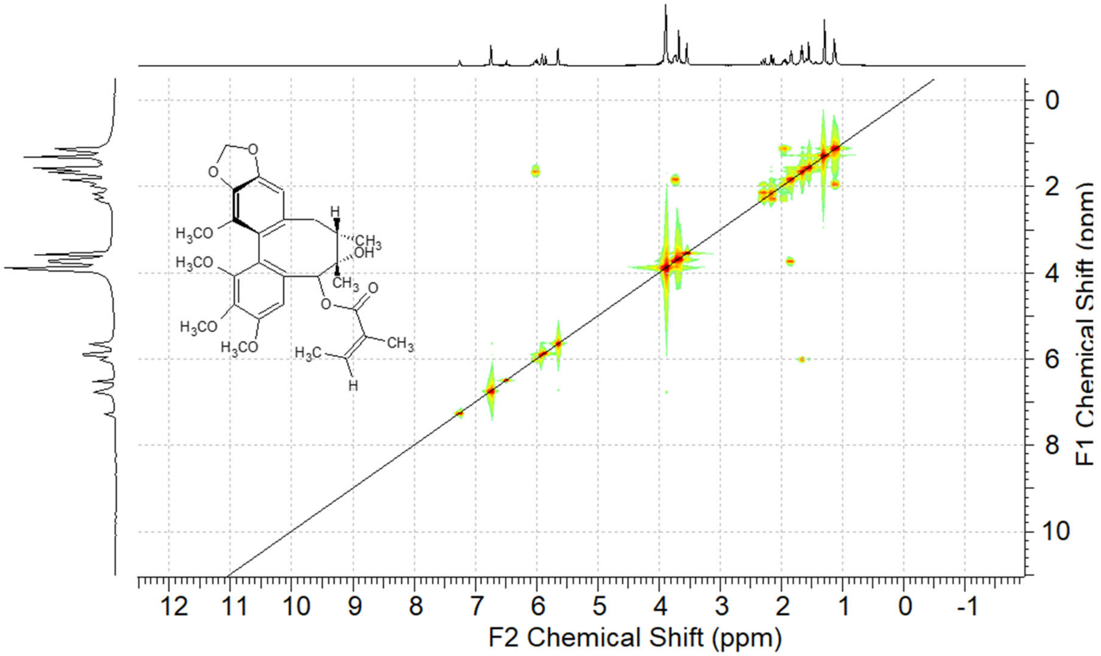

Figure S23.NOESY NMR spectrum of compound **3** in $CDCl_3$

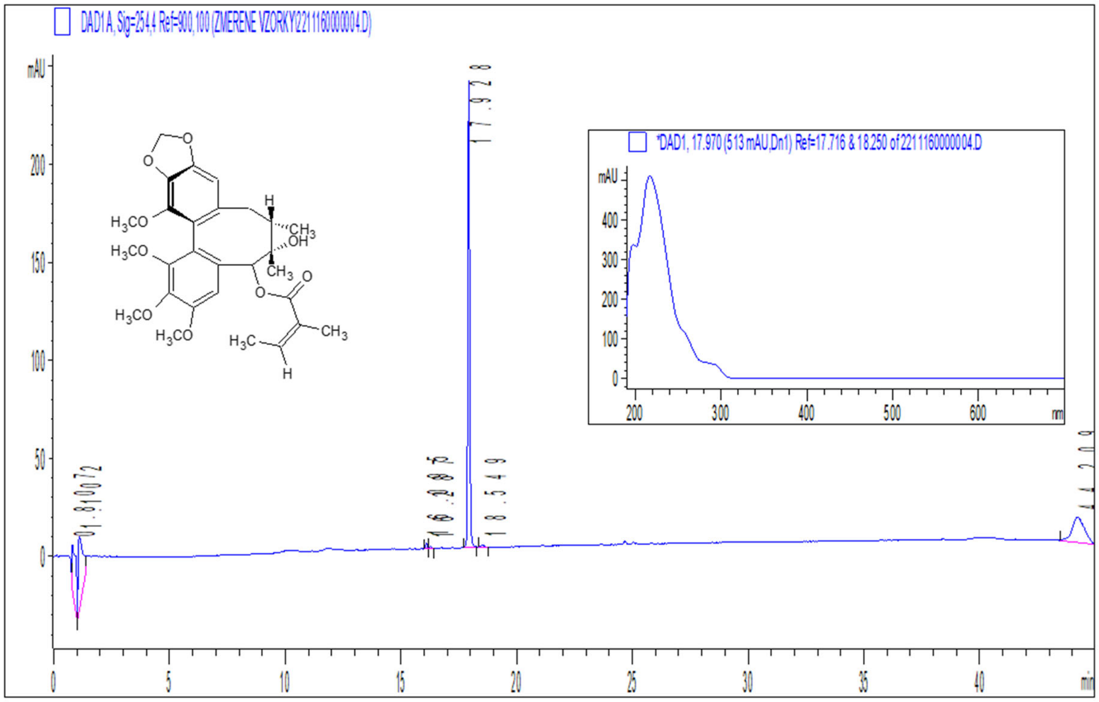

Figure S24. HPLC-DAD chromatogram of compound **3**

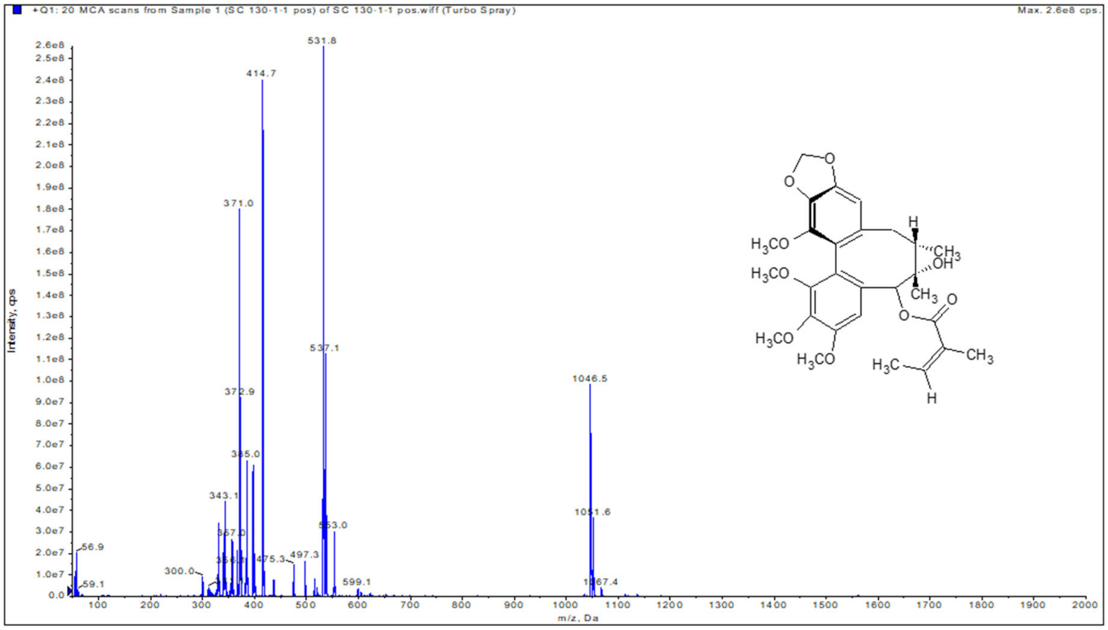

Figure S25. LR-MS spectrum of compound **3**

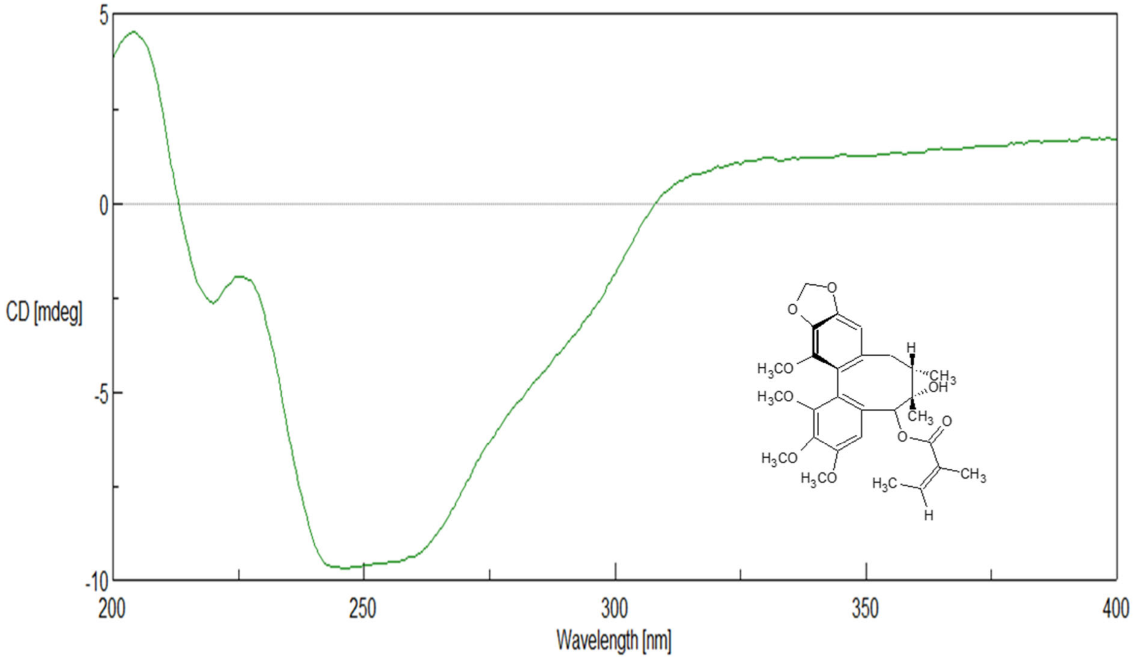

Figure S26. CD spectrum of compound **3** in methanol (c=1 mg/mL)

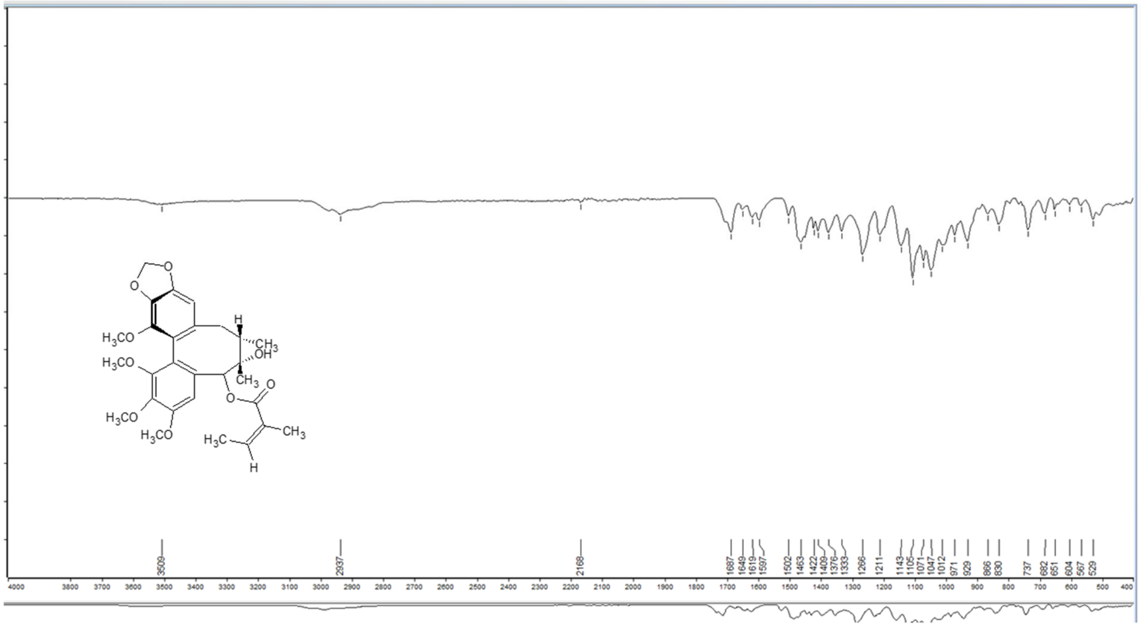

Figure S27. IR spectrum of compound **3**

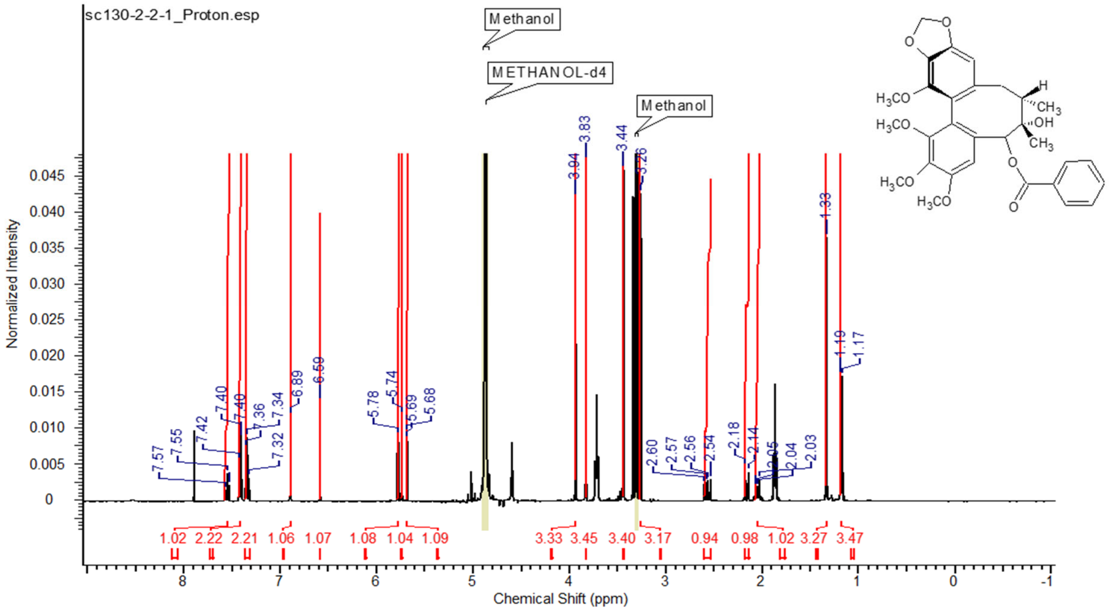

Figure S28. ^{1}H NMR spectrum of compound **4** in CD_3OD

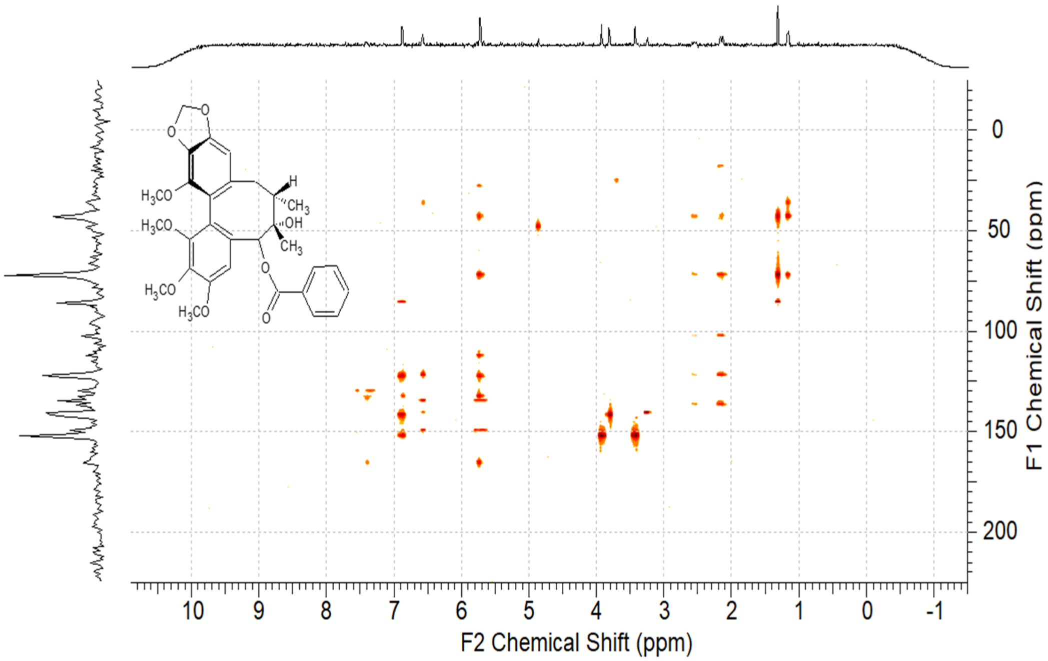

Figure S29. HMBC NMR spectrum of compound **4** in CD_3OD

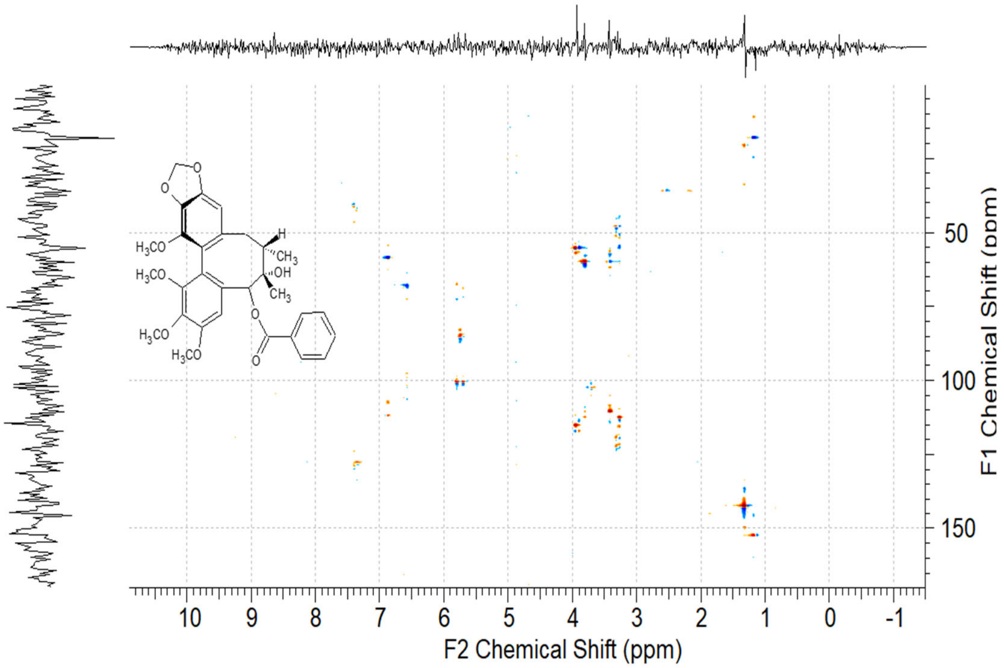

Figure S30. HSQC NMR spectrum of compound **4** in CD_3OD

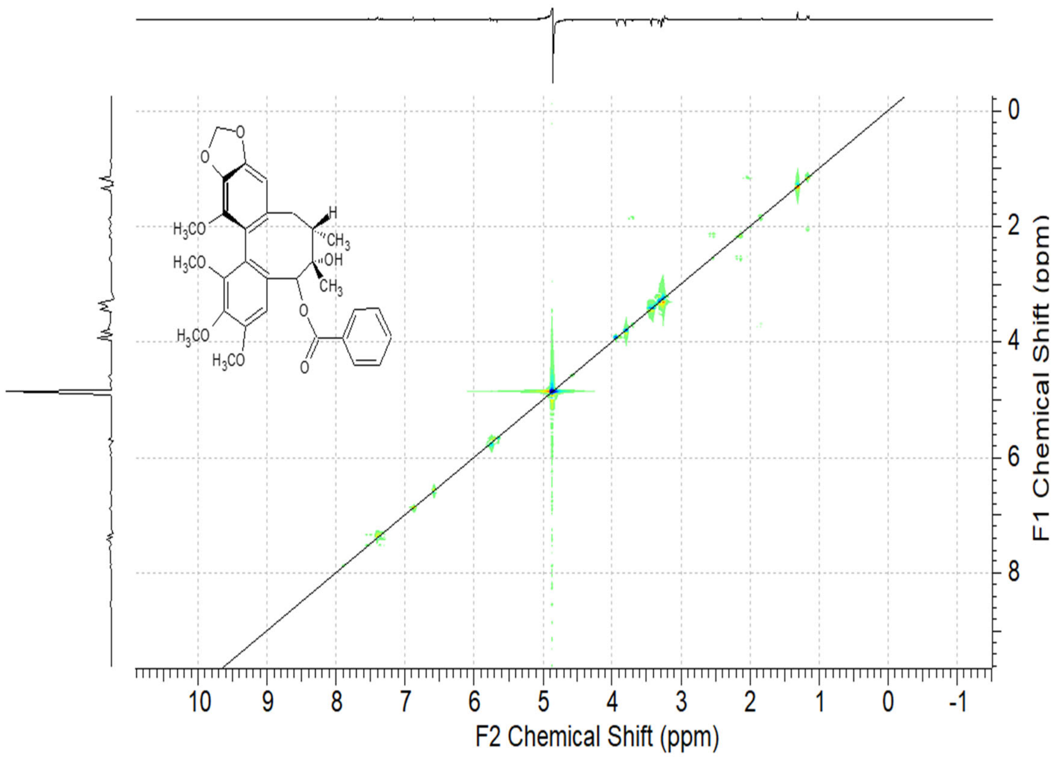

Figure S31. COSY NMR spectrum of compound **4** in CD_3OD

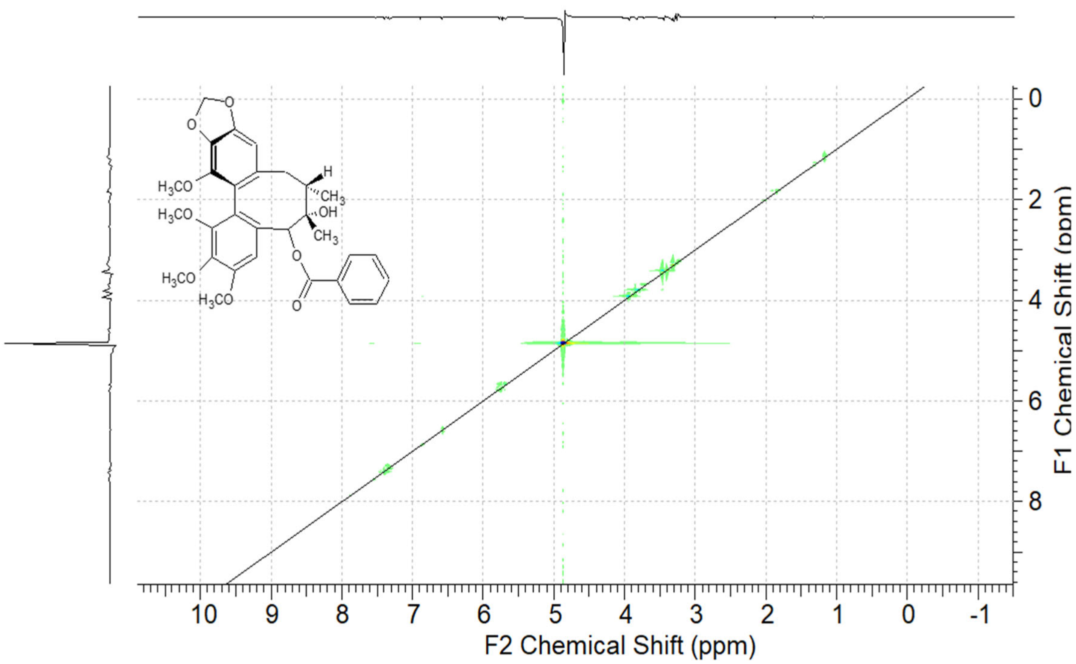

Figure S32. NOESY NMR spectrum of compound **4** in CD_3OD

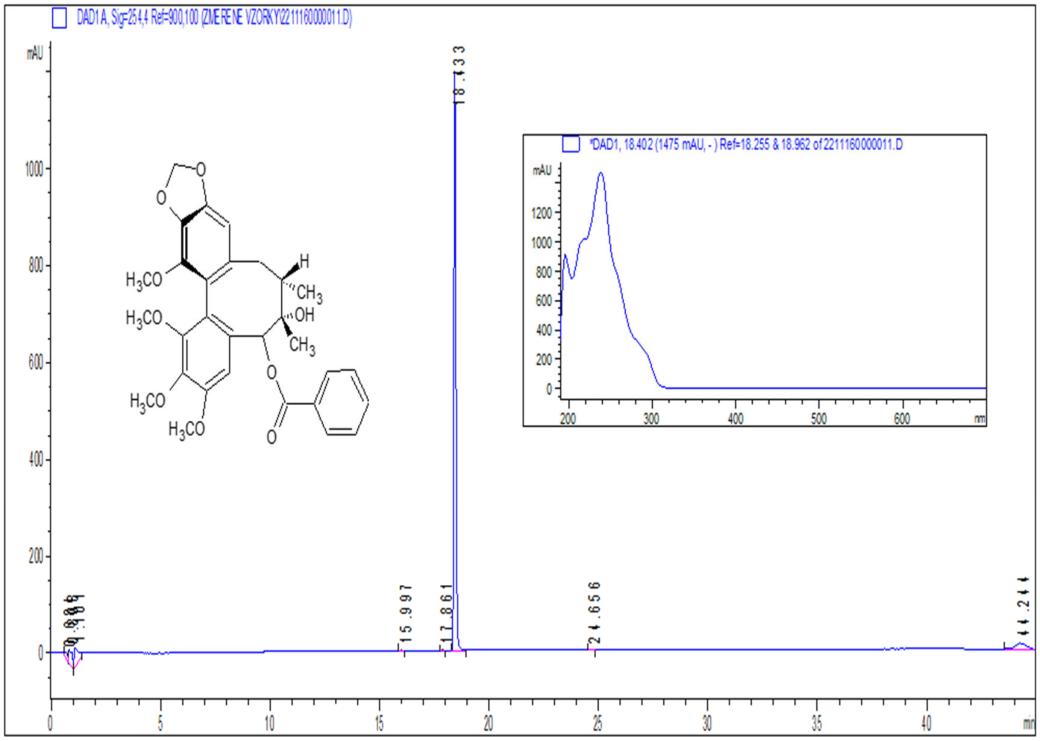

Figure S33. HPLC-DAD chromatogram of compound **4**

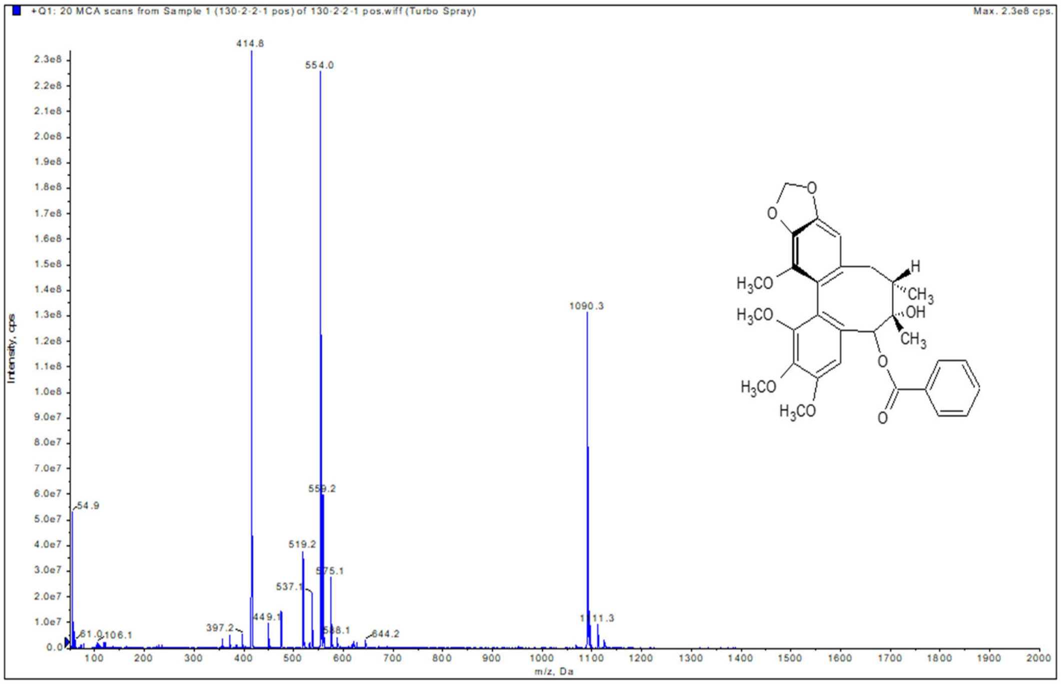

Figure S34. LR-MS chromatogram of compound **4**

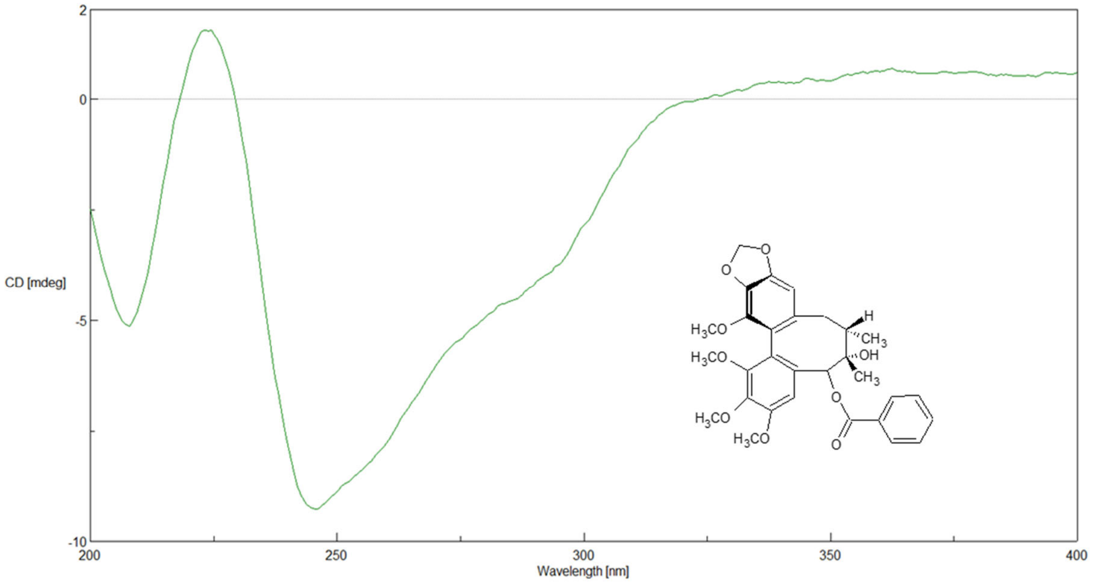

Figure S35. CD spectrum of compound **4** in methanol (c= 1 mg/mL)

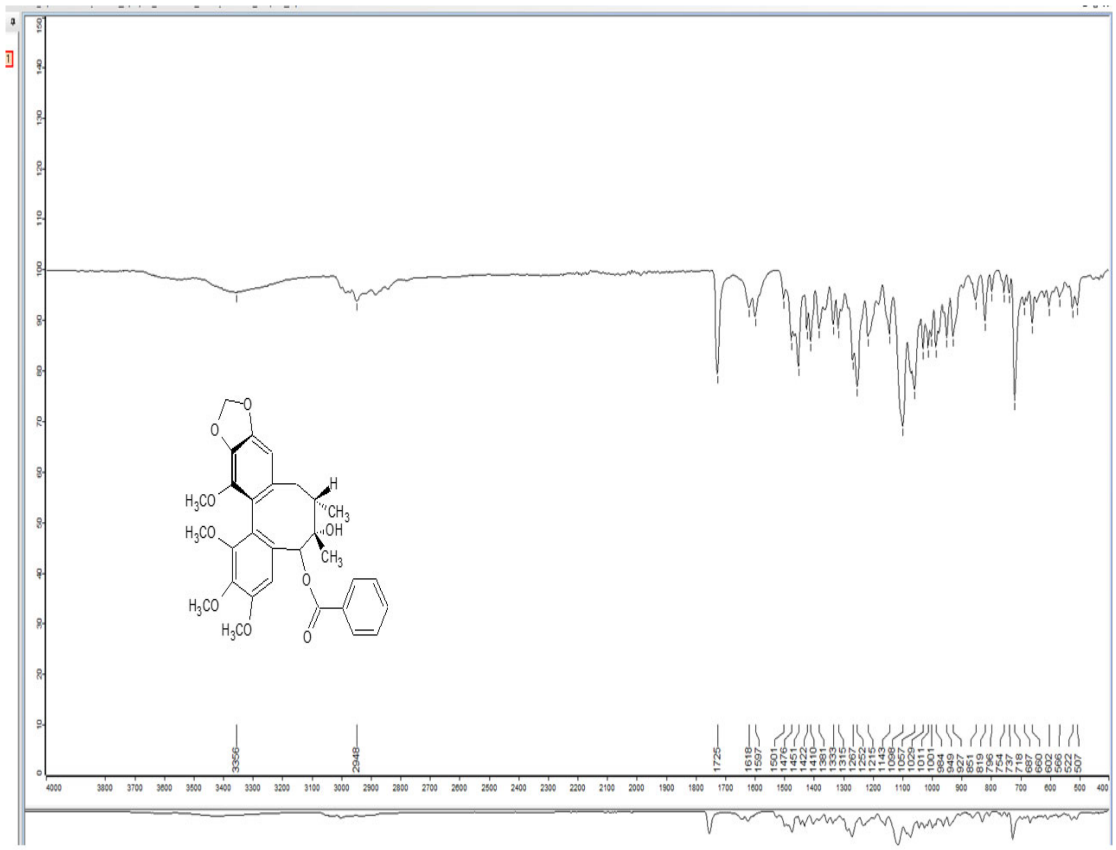

Figure S36. IR spectrum of compound **4**

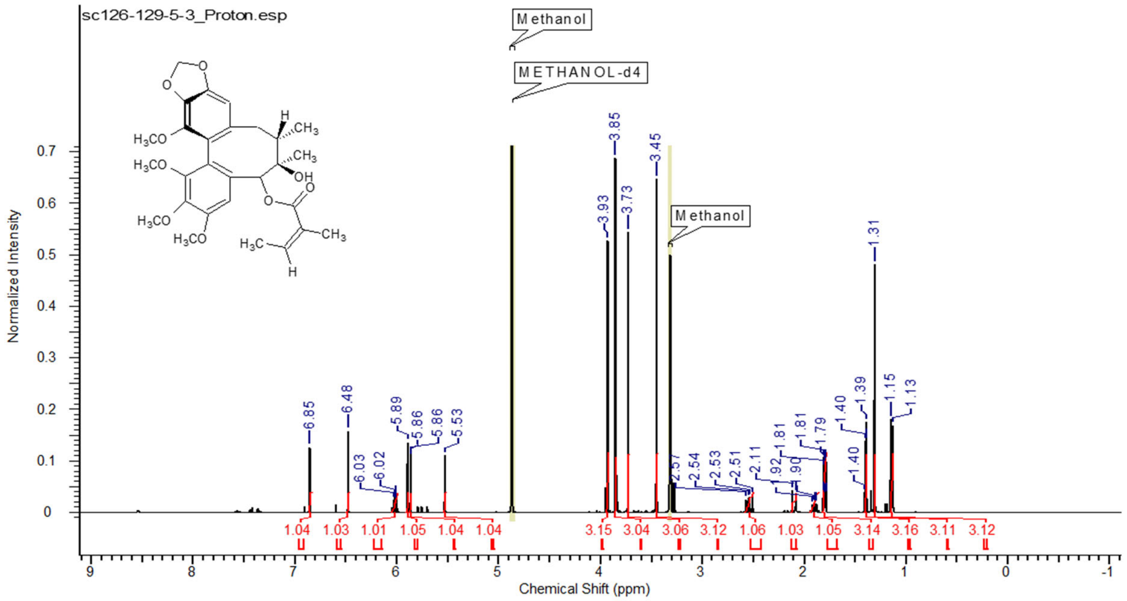

Figure S37. ^{1}H NMR spectrum of compound **5** in CD3OD

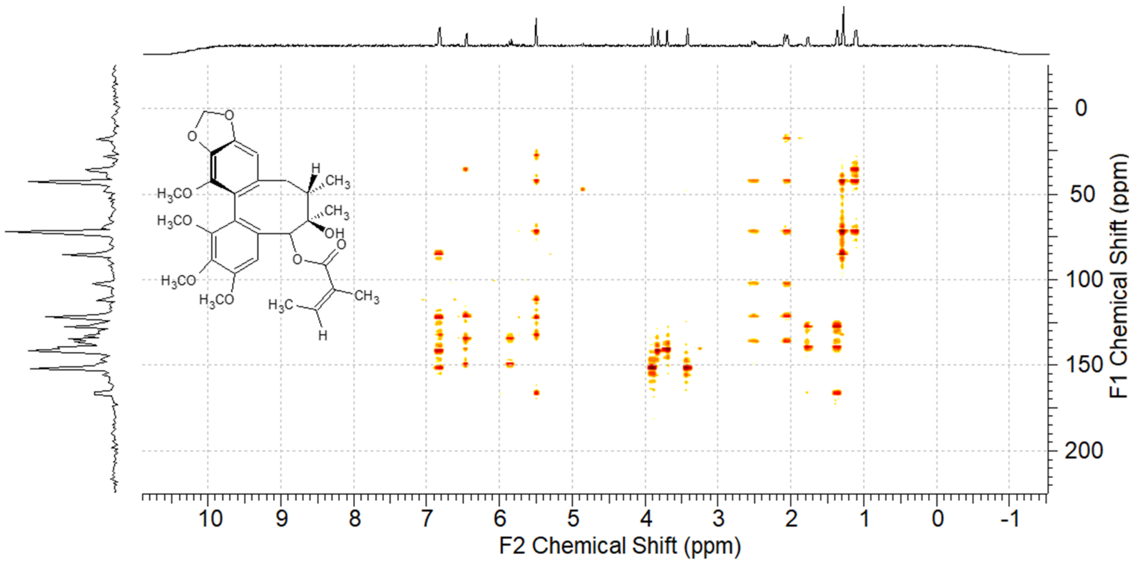

Figure S38. HMBC NMR spectrum of compound **5** in CD3OD

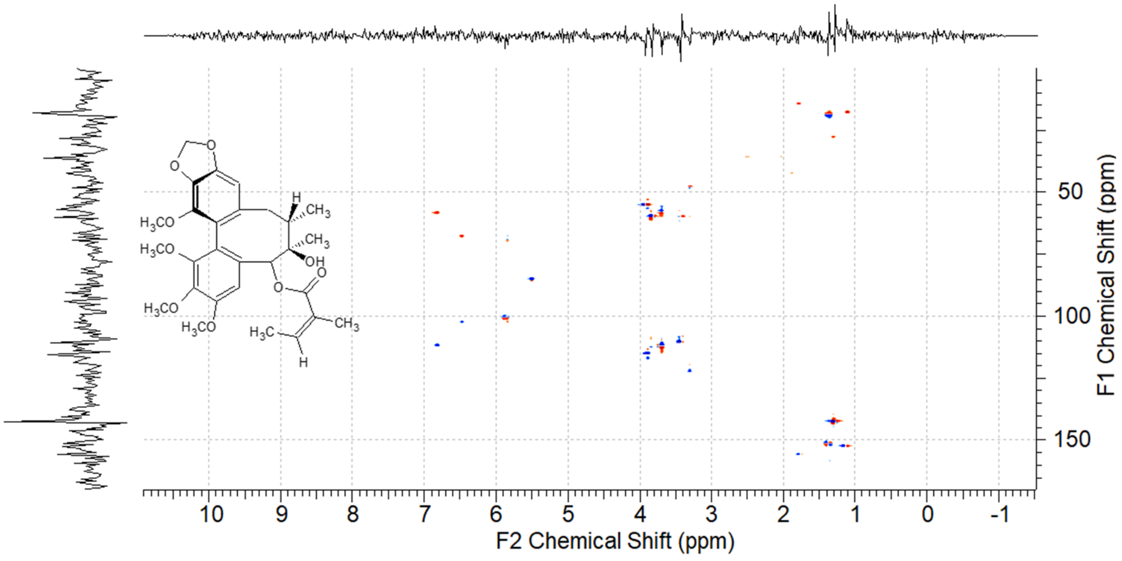

Figure S39. HSQC NMR spectrum of compound **5** in CD_3OD

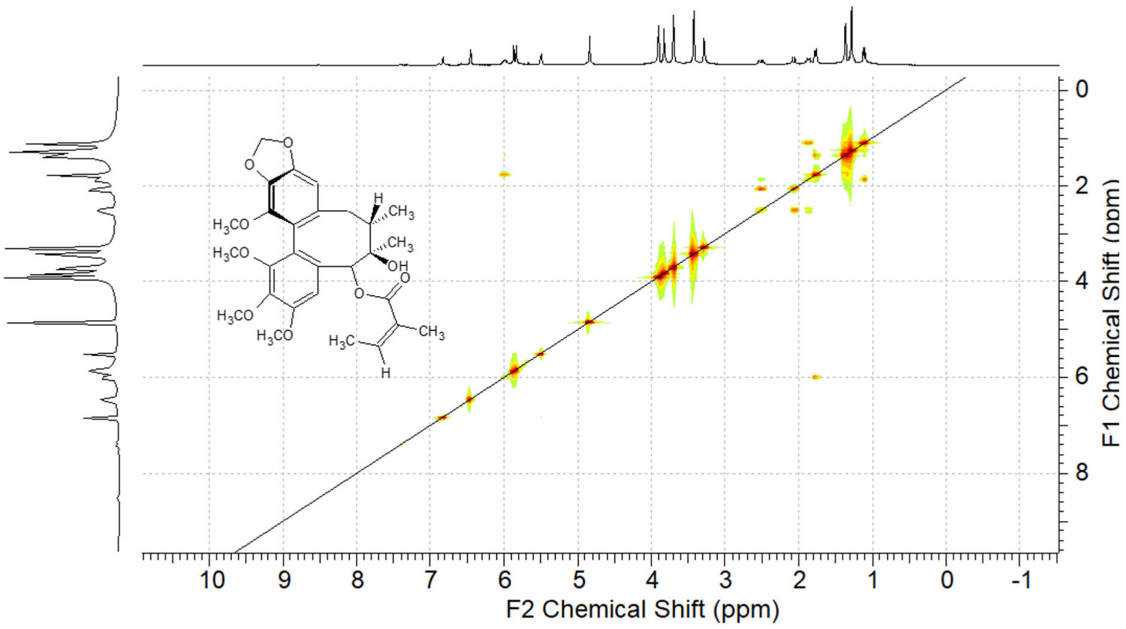

Figure S40. COSY NMR spectrum of compound **5** in CD_3OD

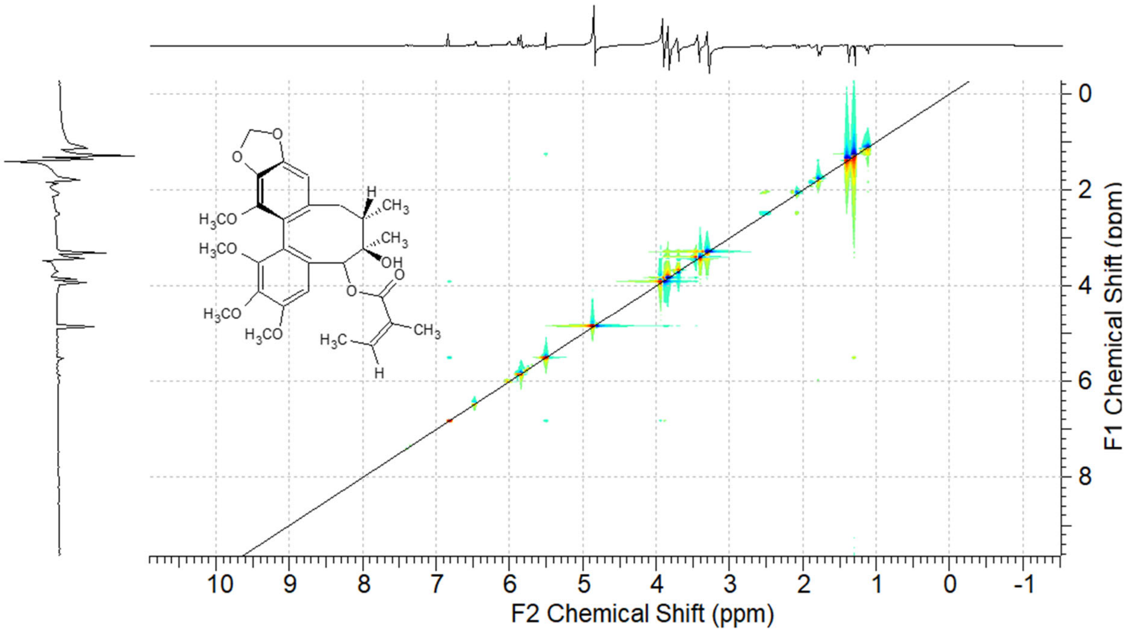

Figure S41. NOESY NMR spectrum of compound **5** in CD_3OD

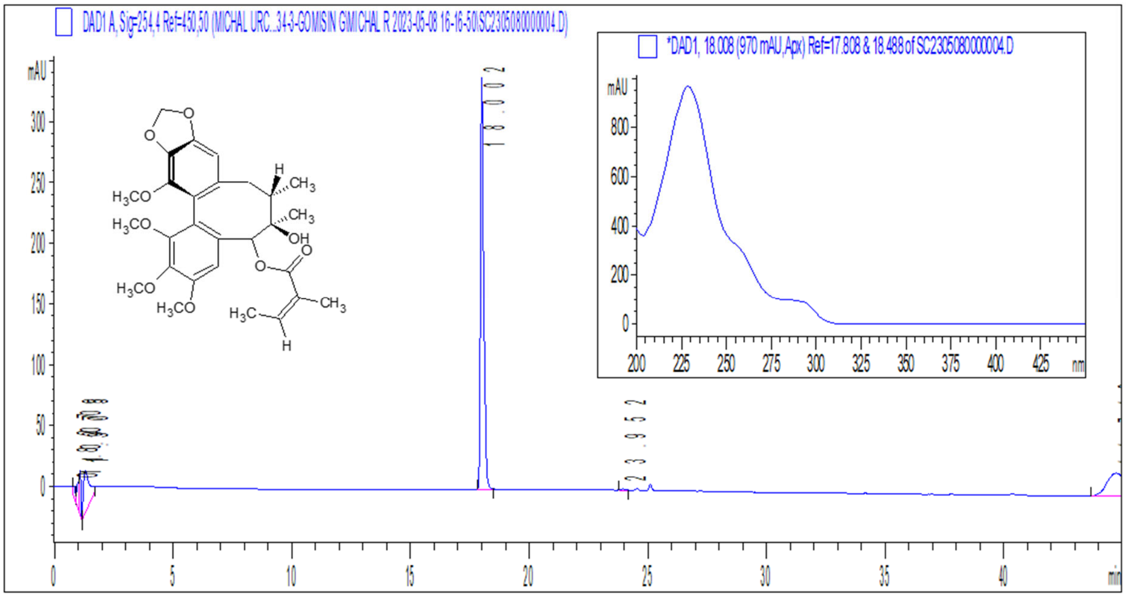

Figure S42. HPLC-DAD chromatogram of compound **5**

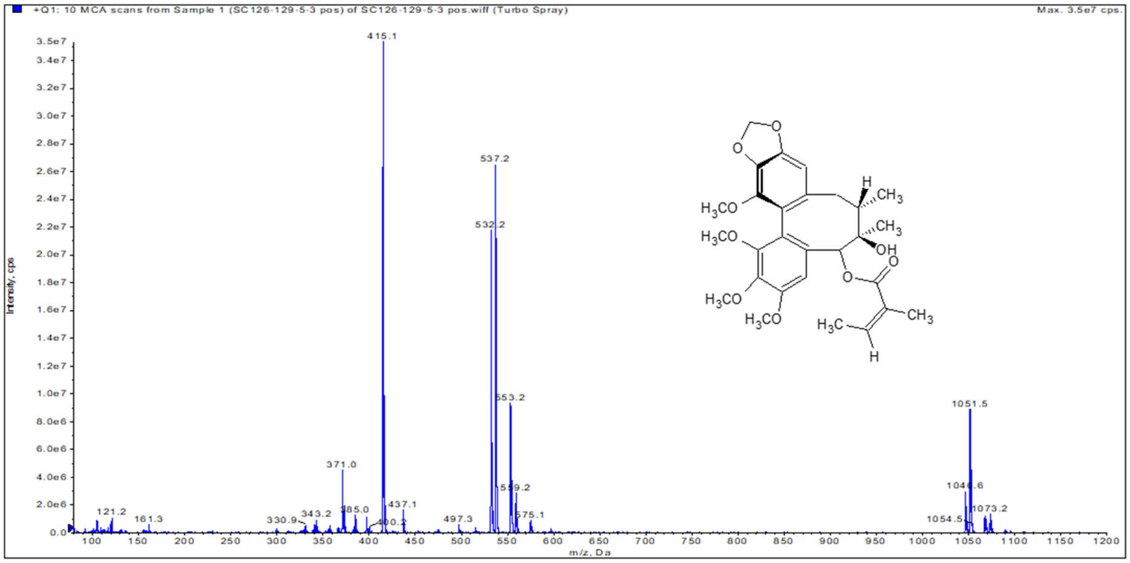

Figure S43. LR-MS spectrum of compound **5**

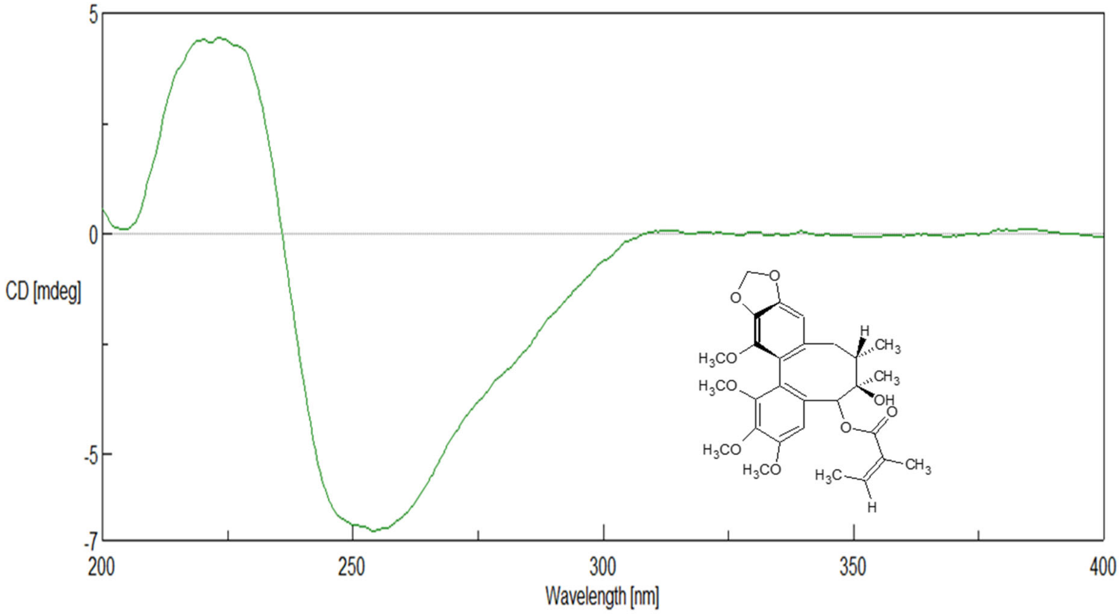

Figure S44. CD spectrum of compound **5** in methanol (c=1 mg/mL))

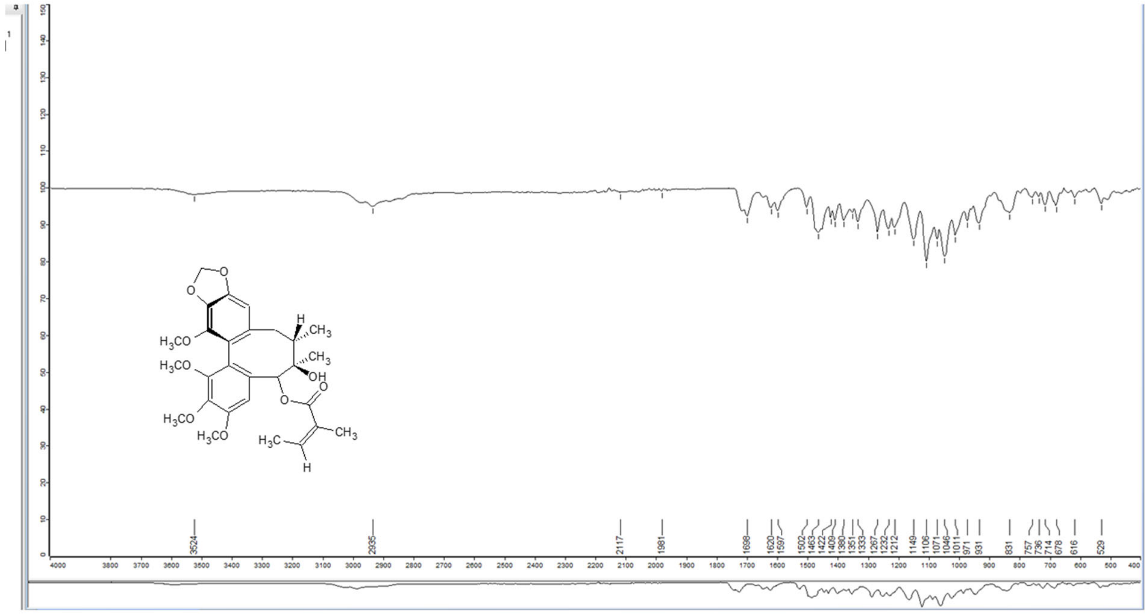

Figure S45. IR spectrum of compound **5**

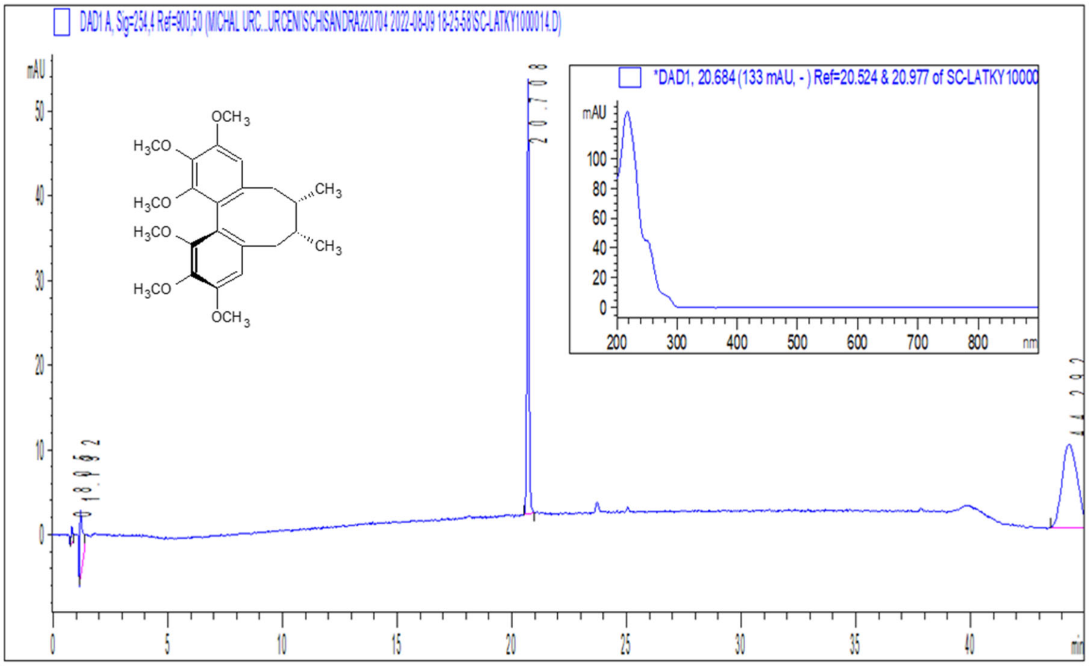

Figure S46. HPLC-DAD chromatogram of common injection of compound **6** with standard of (+)-deoxyschisandrin; UV spectrum of peak corresponding to (+)-deoxyschisandrin.

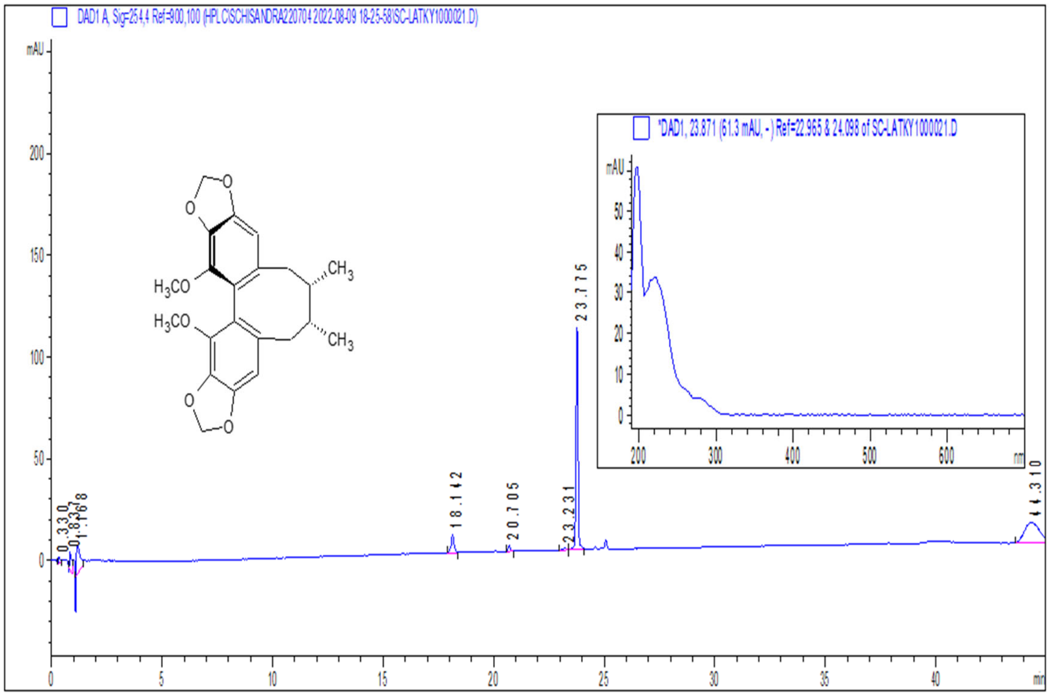

Figure S47. HPLC-DAD chromatogram of common injection of (−)-wuweizisu C with standard of (−)-wuweizisu C; UV spectrum of peak corresponding to (−)-wuweizisu C.

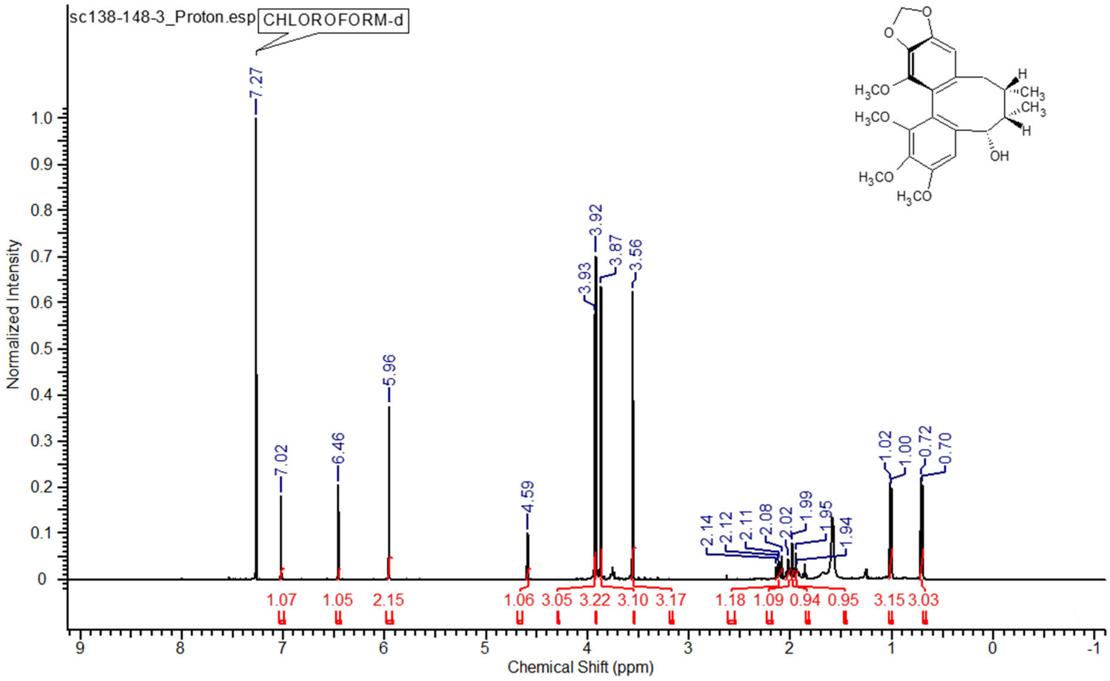

Figure S48. ^{1}H NMR spectrum of epigomisin O in $CDCl_3$

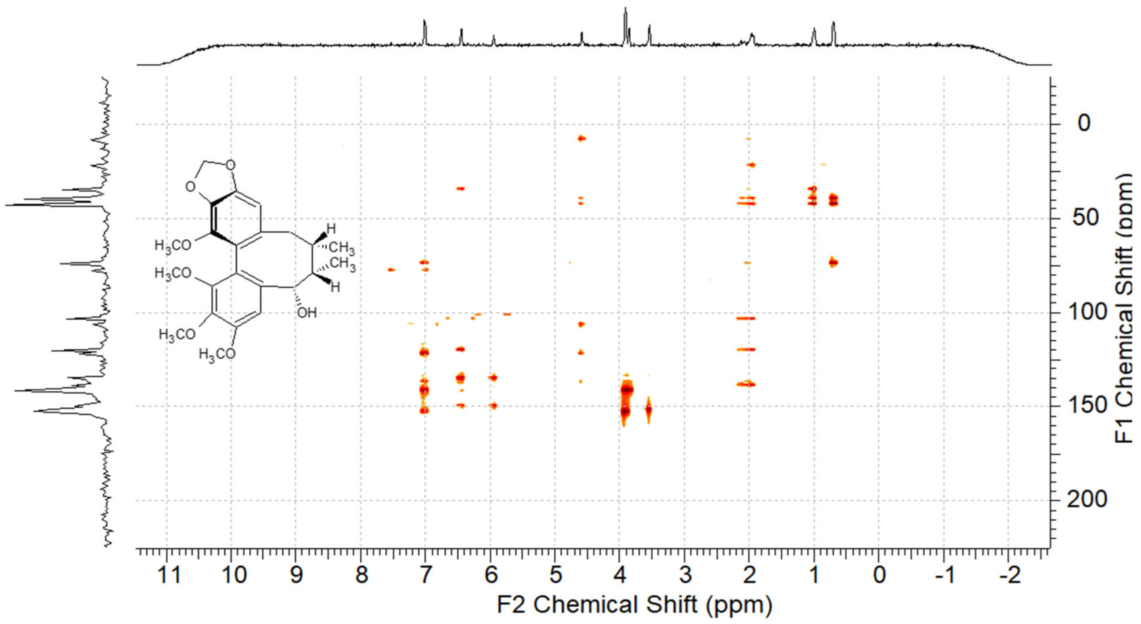

Figure S49. HMBC NMR spectrum of epigomisin O in CDCl3

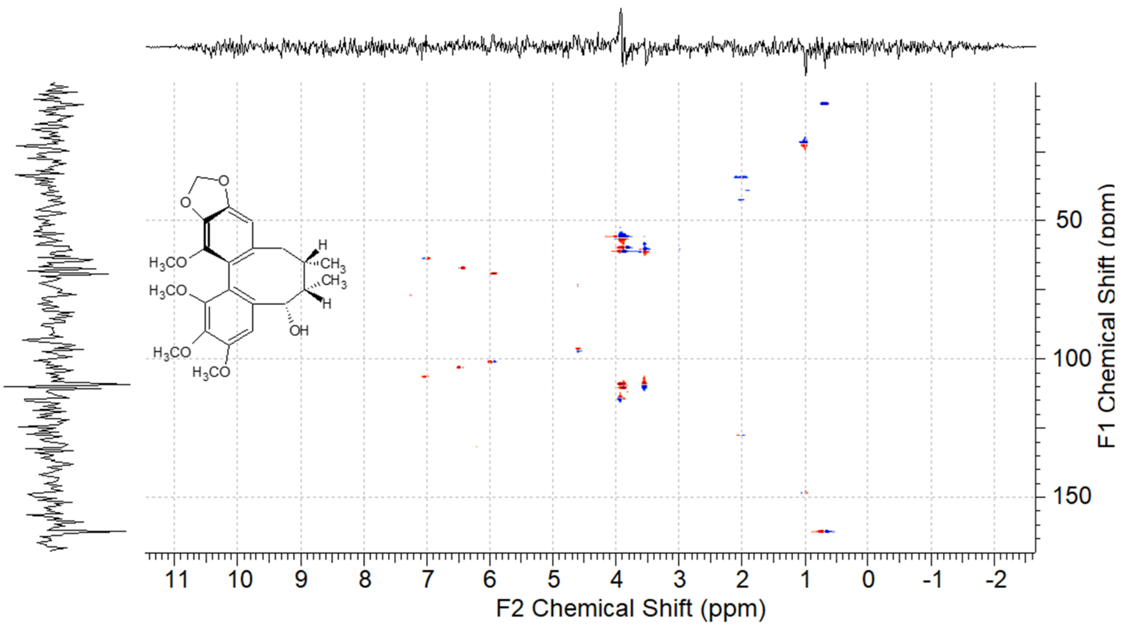

Figure S50. HSQC NMR spectrum of epigomisin O in CDCl3

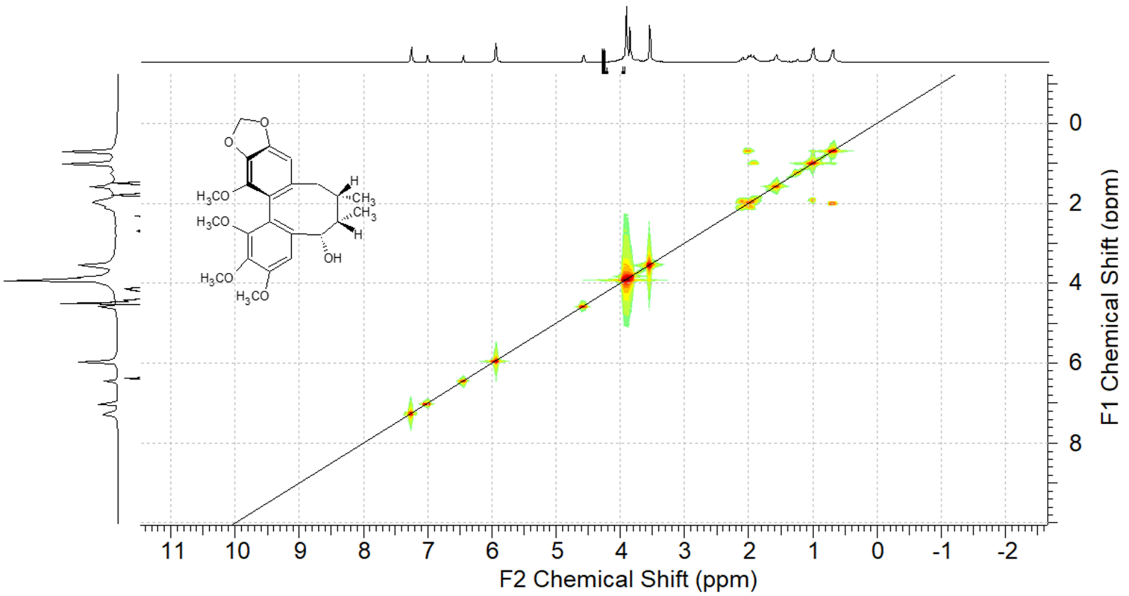

Figure S51. COSY NMR spectrum of epigomisin O in CDCl3

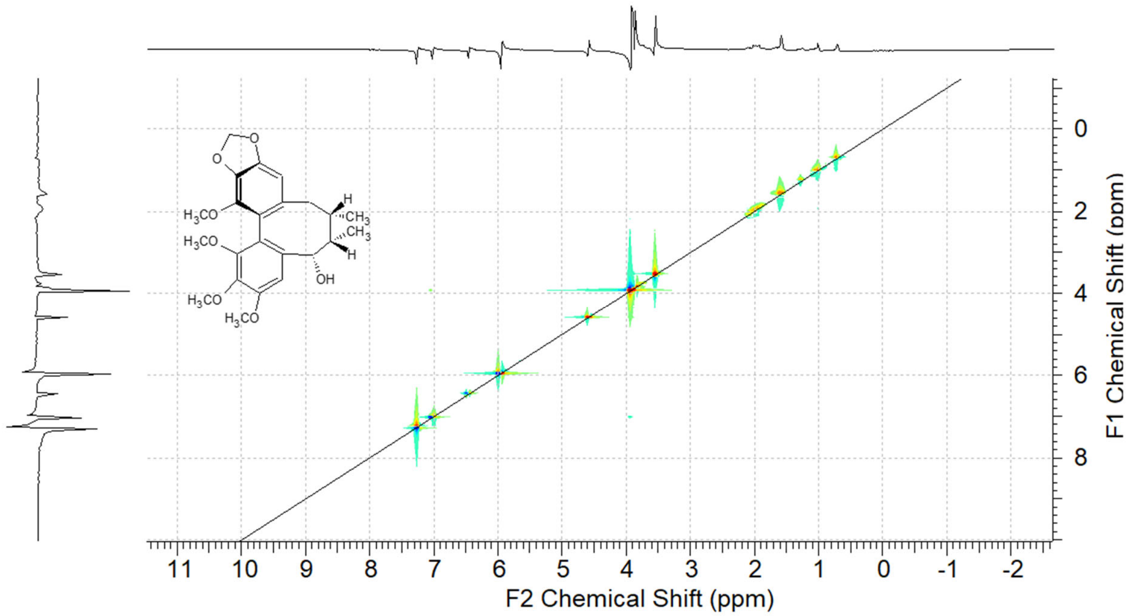

Figure S52. NOESY NMR spectrum of epigomisin O in CDCl3

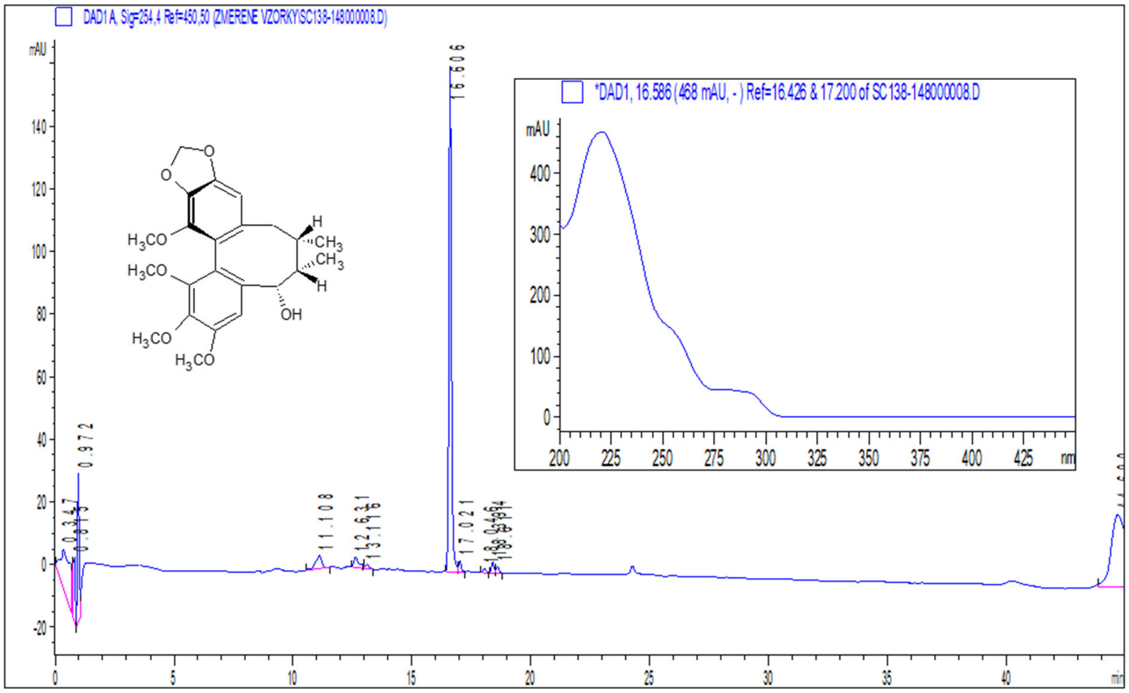

Figure S53. HPLC-DAD chromatogram of epigomisin O

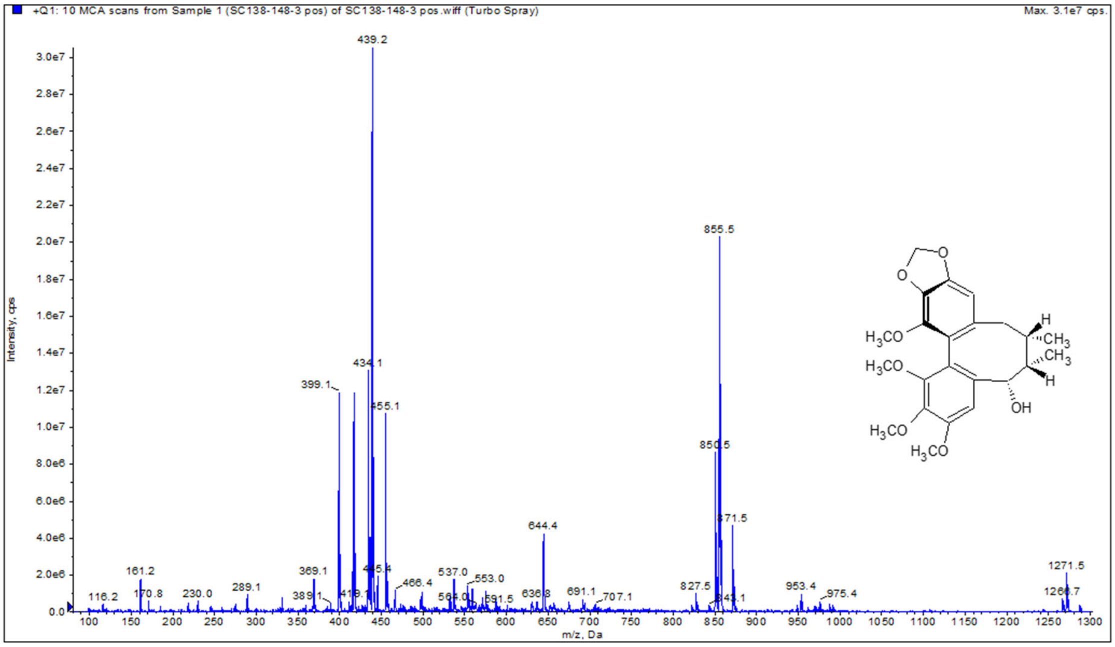

Figure S54. LR-MS spectrum of epigomisin O

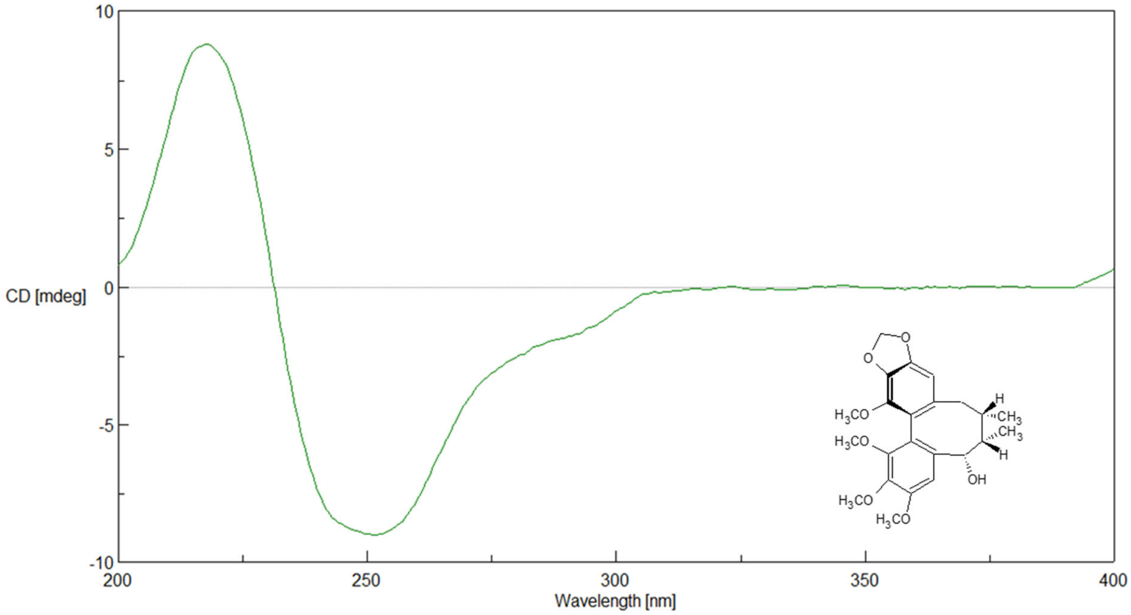

Figure S55. CD spectrum of epigomisin O in methanol (c=1 mg/mL))

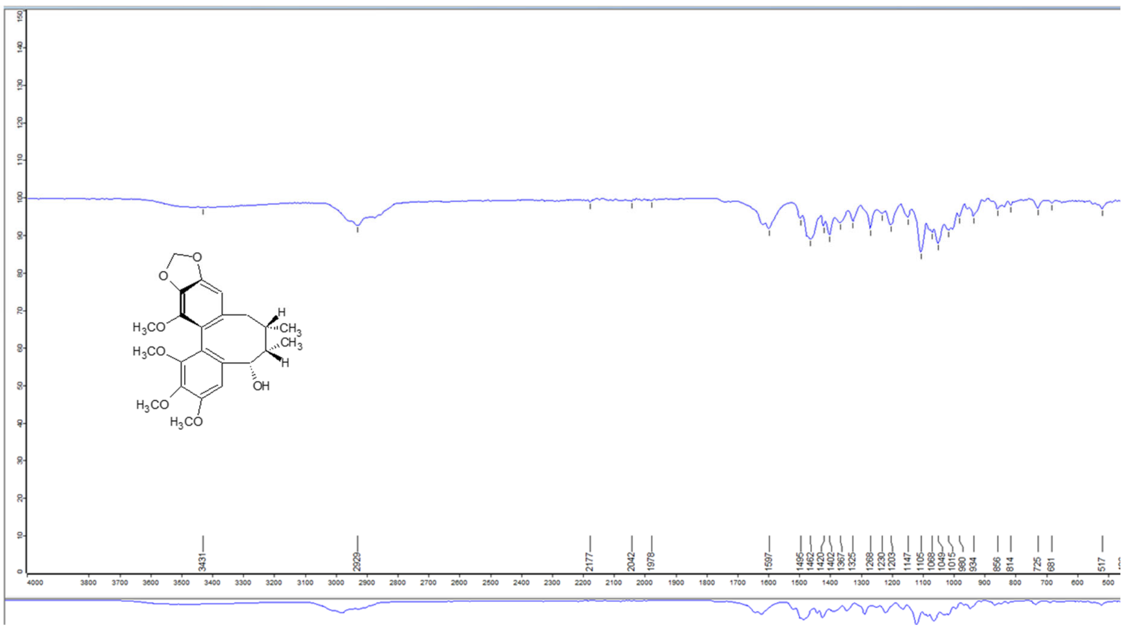

Figure S56. IR spectrum of epigomisin O

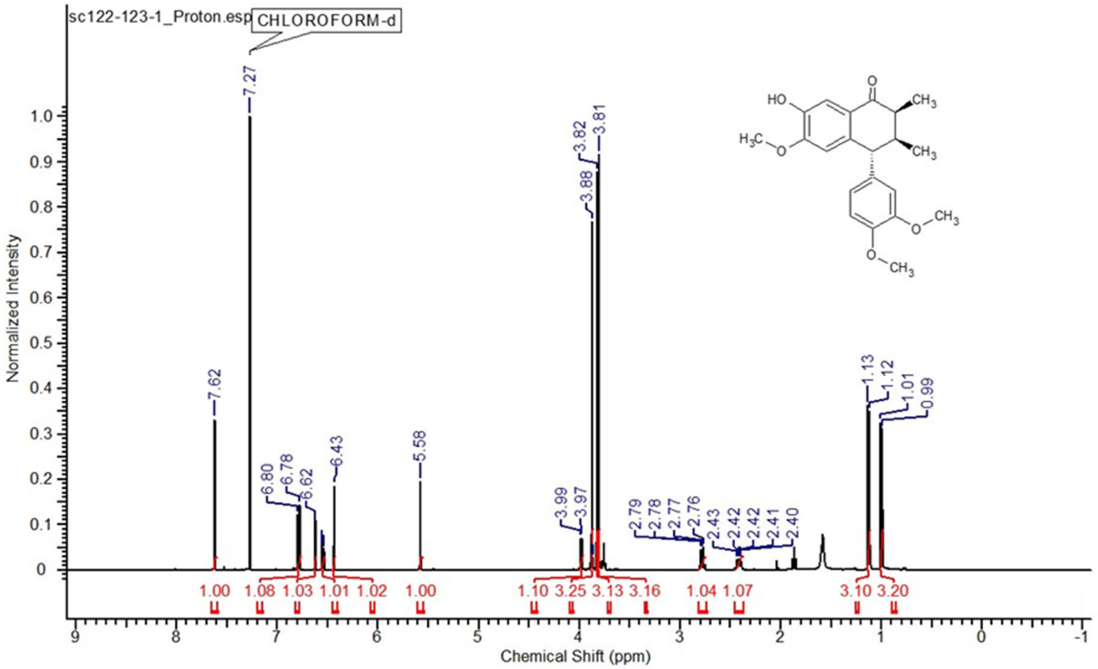

Figure S57. ^{1}H NMR spectrum of arisantetralone C in $CDCl_3$

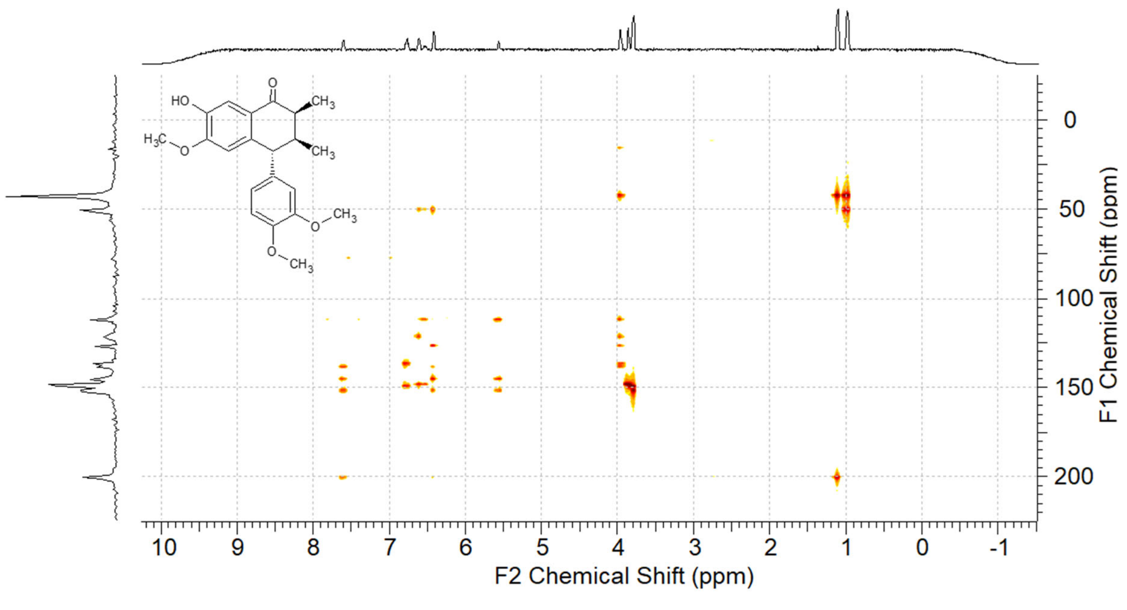

Figure S58. HMBC NMR spectrum of arisantetralone C in $CDCl_3$

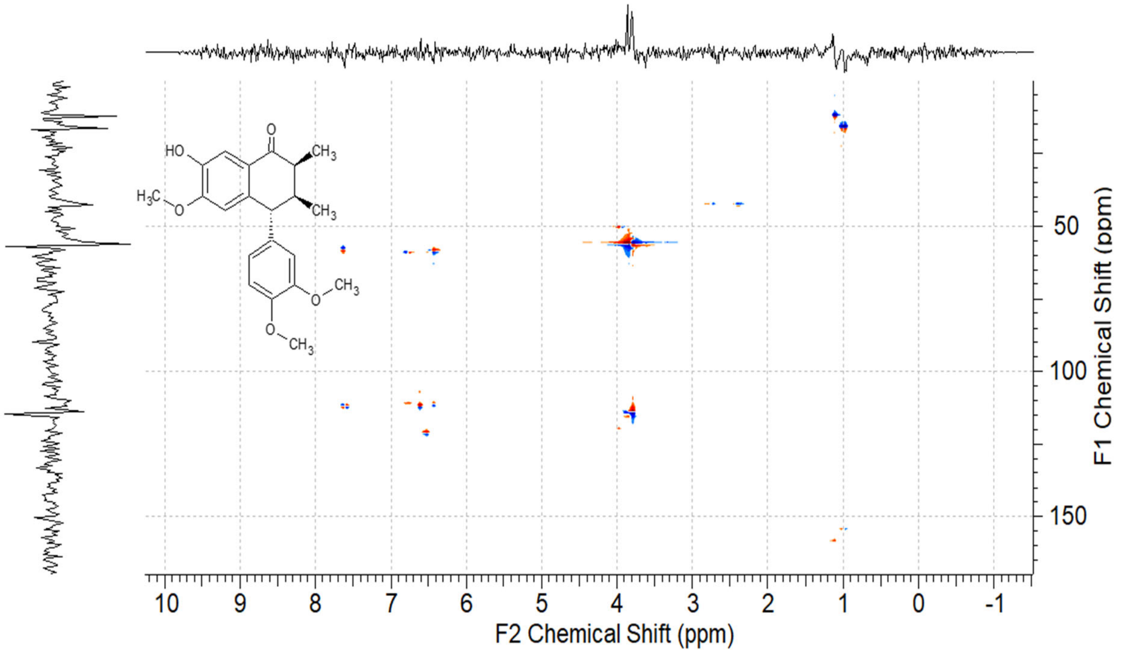

Figure S59. HSQC NMR spectrum of arisantetralone C in $CDCl_3$

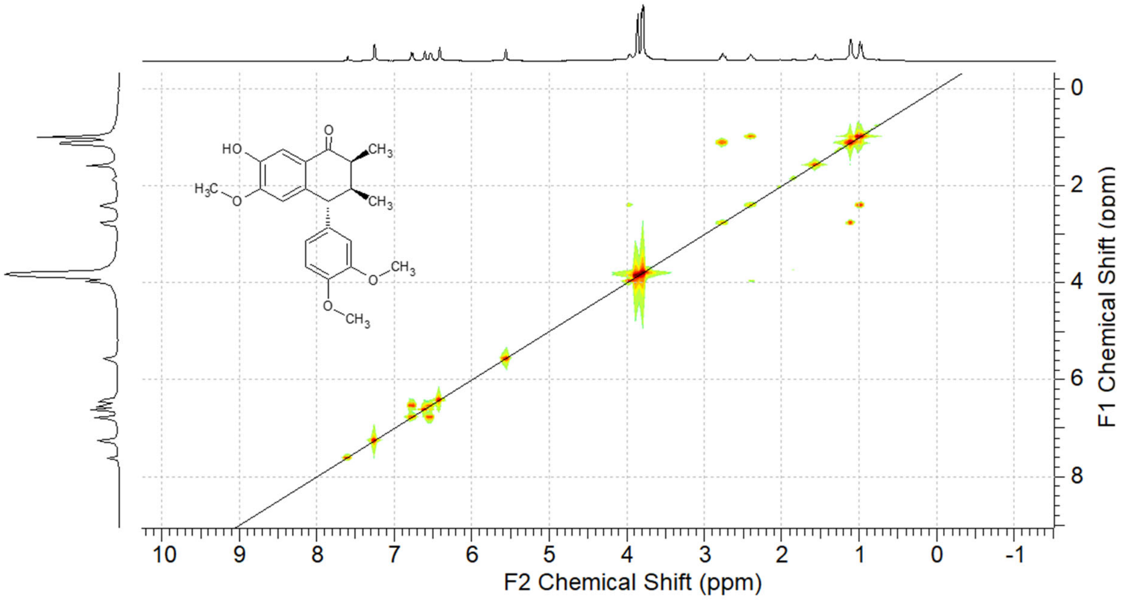

Figure S60. COSY NMR spectrum of arisantetralone C in $CDCl_3$

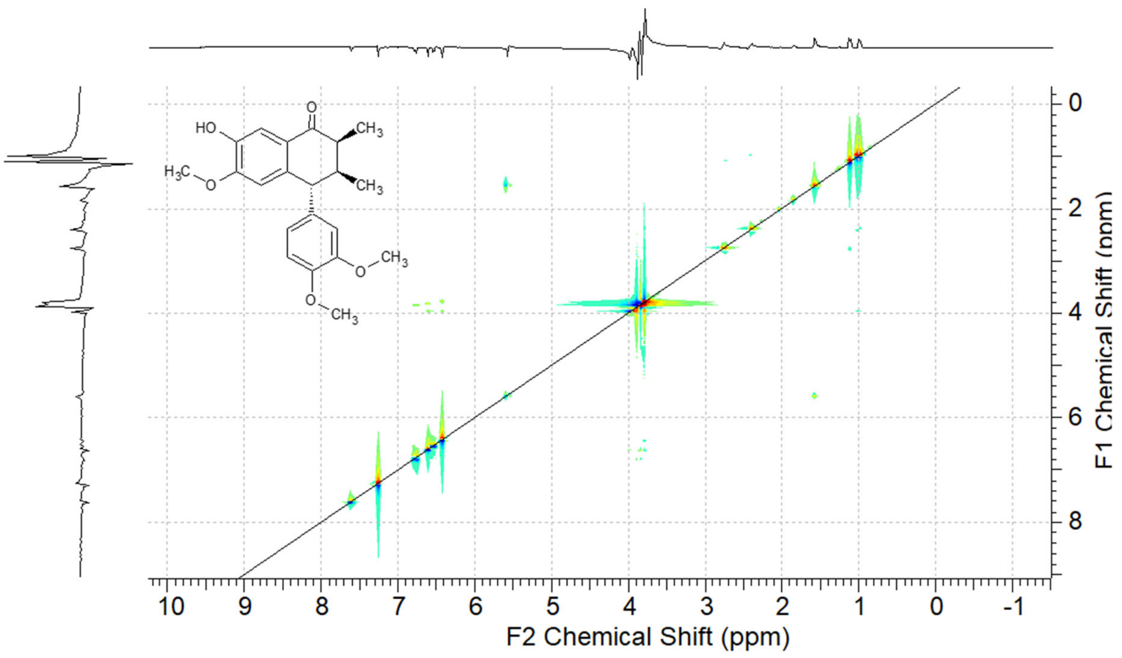

Figure S61. NOESY NMR spectrum of arisantetralone C in $CDCl_3$

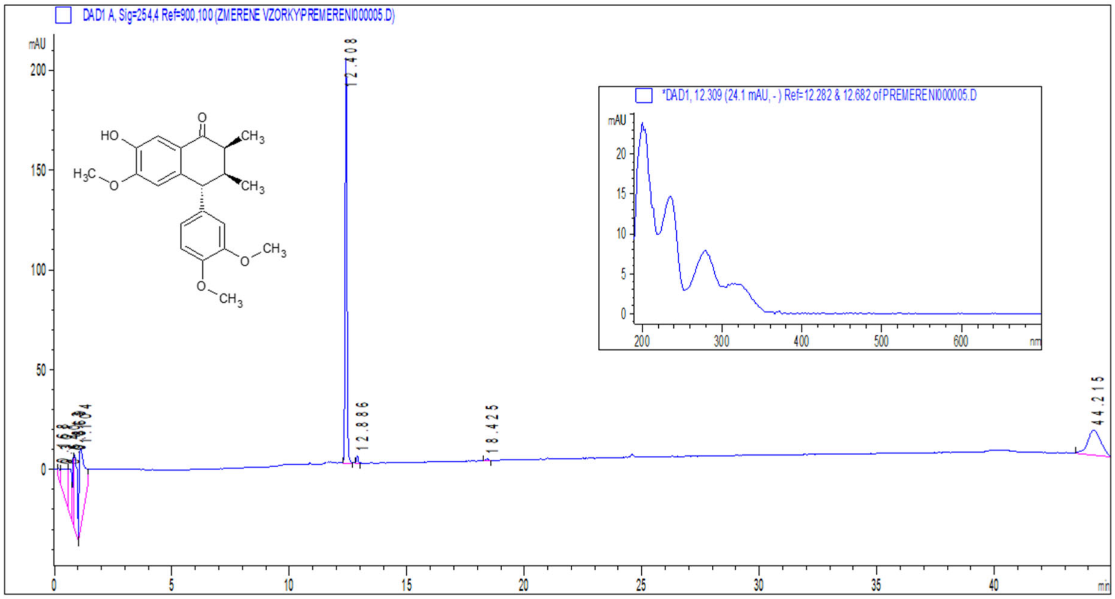

Figure S62. HPLC-DAD chromatogram of arisantetralone C

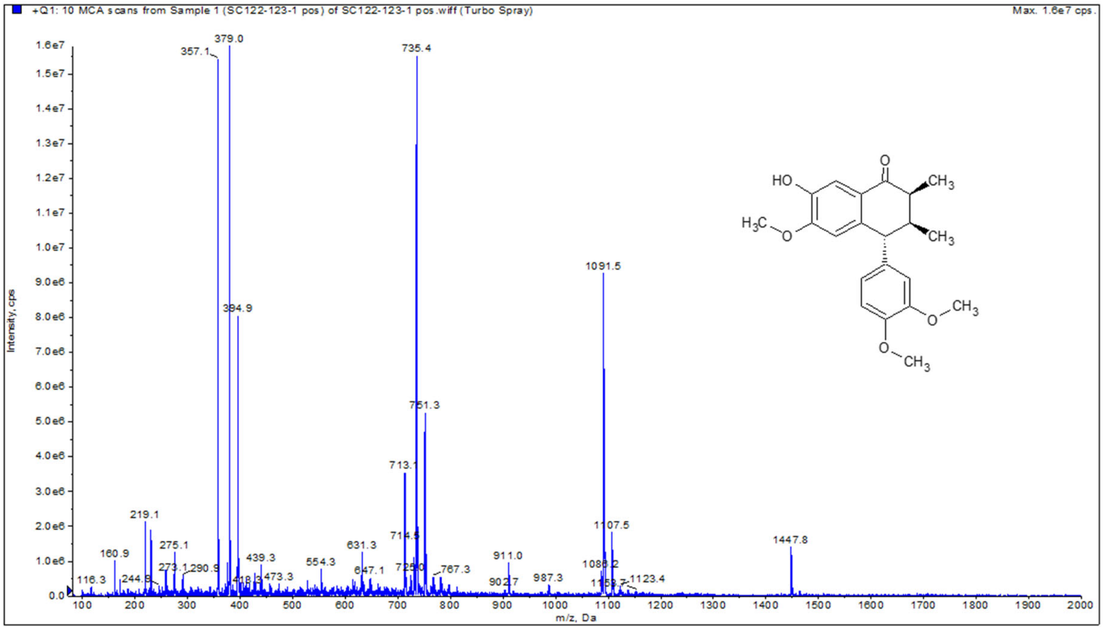

Figure S63. LR-MS spectrum of arisantetralone C

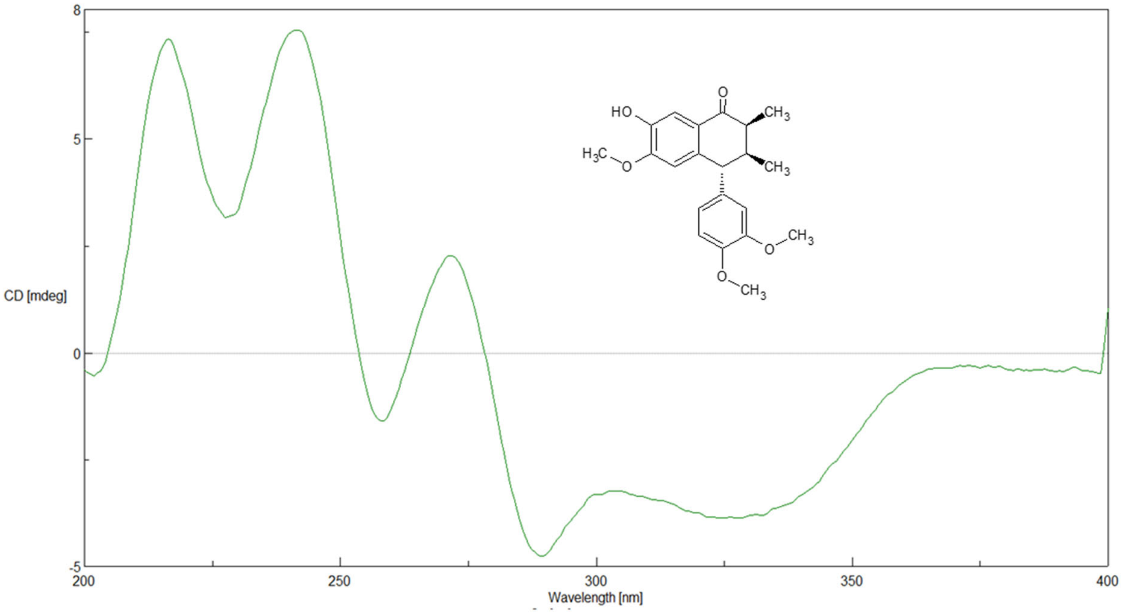

Figure S64. CD spectrum of arisantetralone C in methanol (c =1 mg/mL)

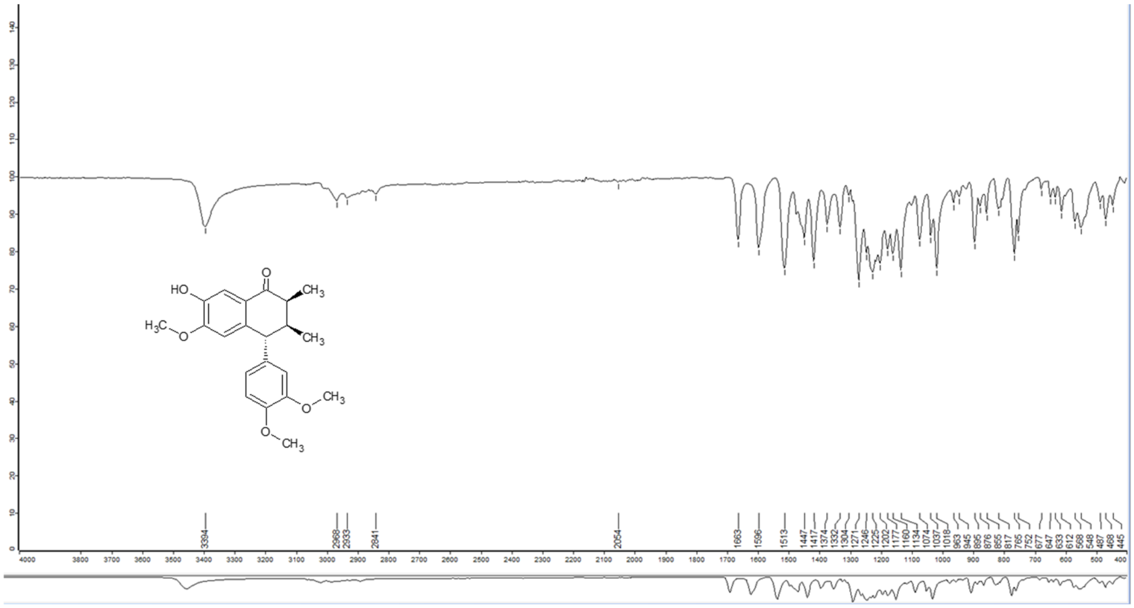

Figure S65. IR spectrum of arisantetralone C

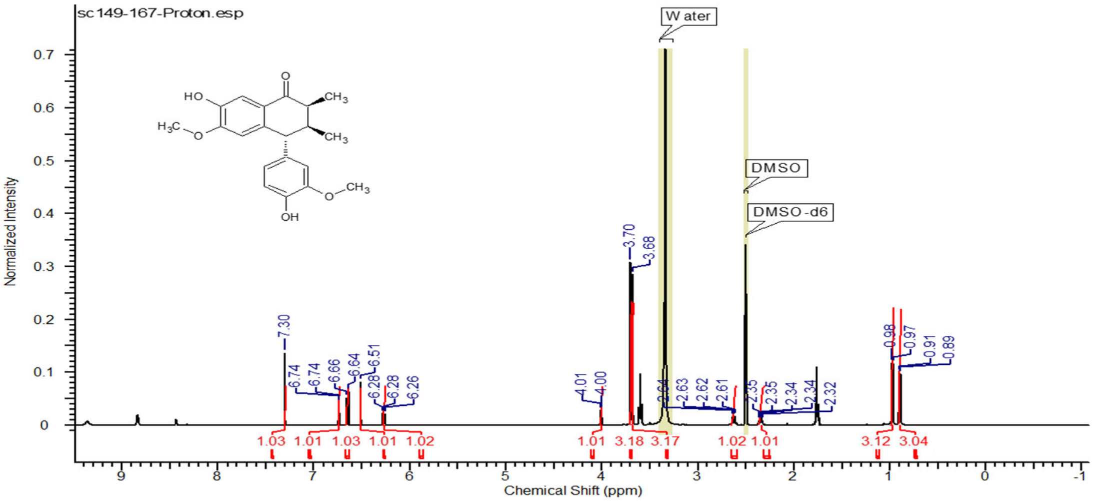

Figure S66. ^{1}H NMR spectrum of arisantetralone A in DMSO-d_6

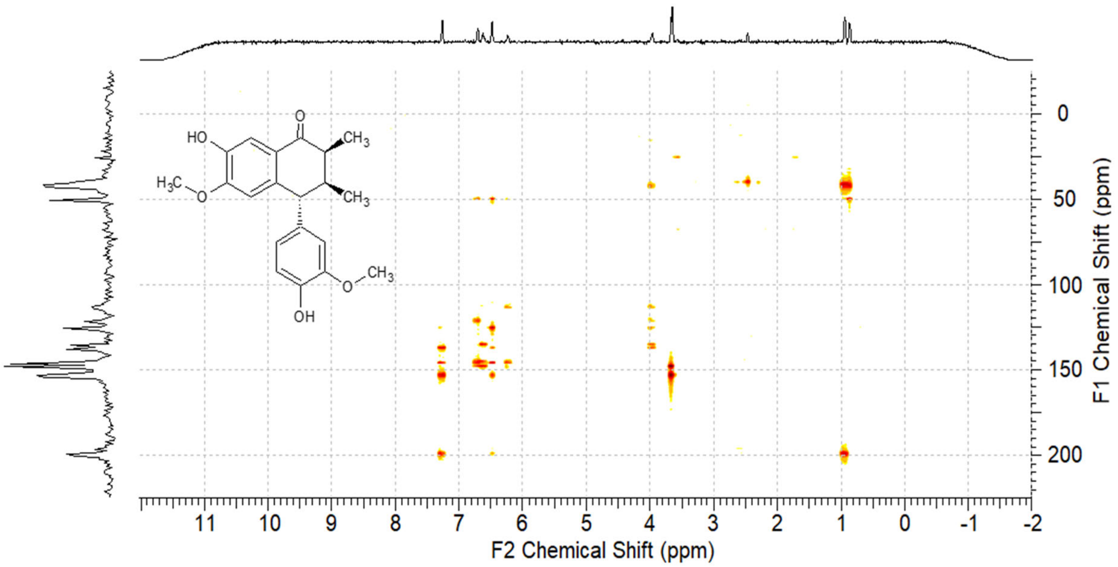

Figure S67. HMBC NMR spectrum of arisantetralone A in DMSO-d_6

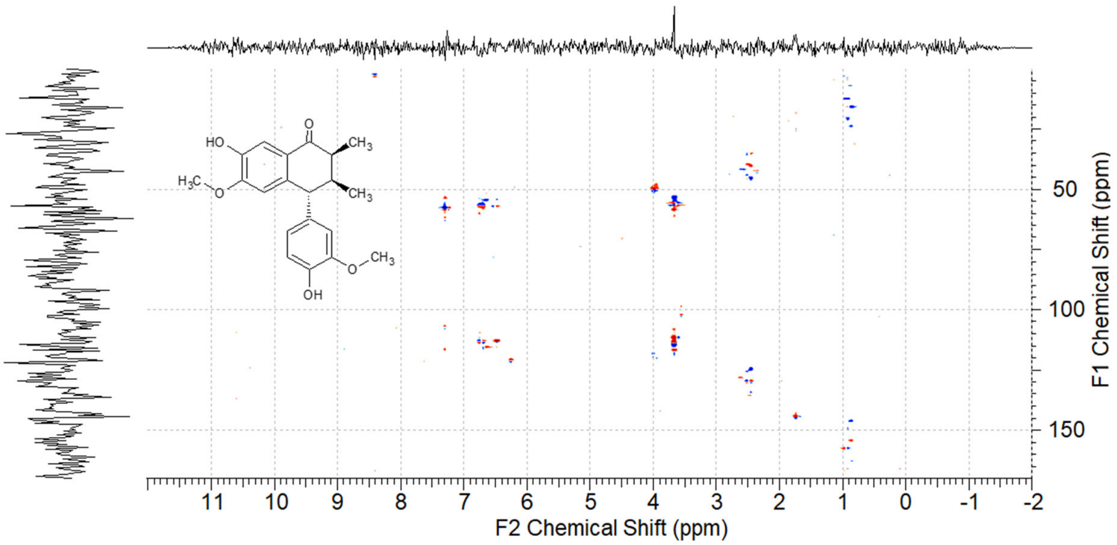

Figure S68. HSQC NMR spectrum of arisantetralone A in DMSO-d_6

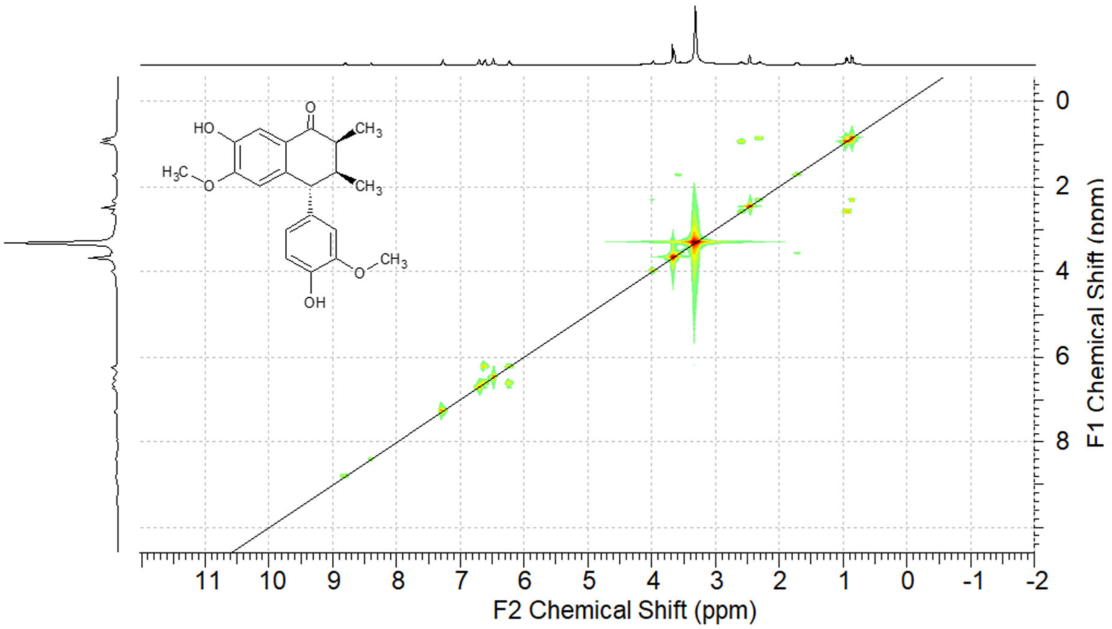

Figure 69. COSY NMR spectrum of arisantetralone A in DMSO-d_6

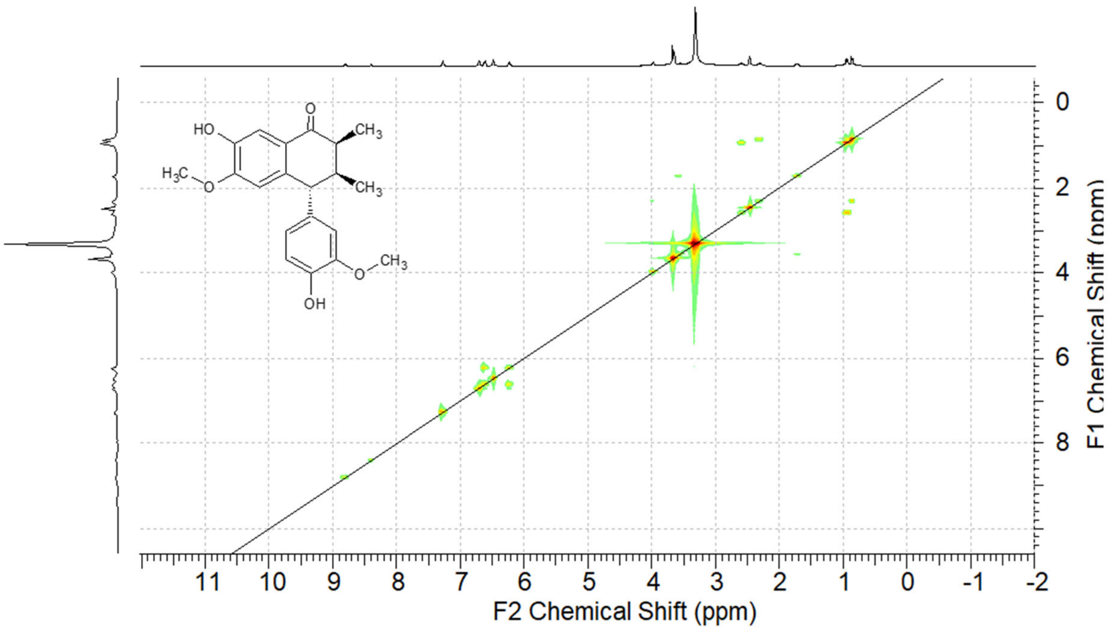

Figure S70. NOESY NMR spectrum of arisantetralone A in DMSO-d_6

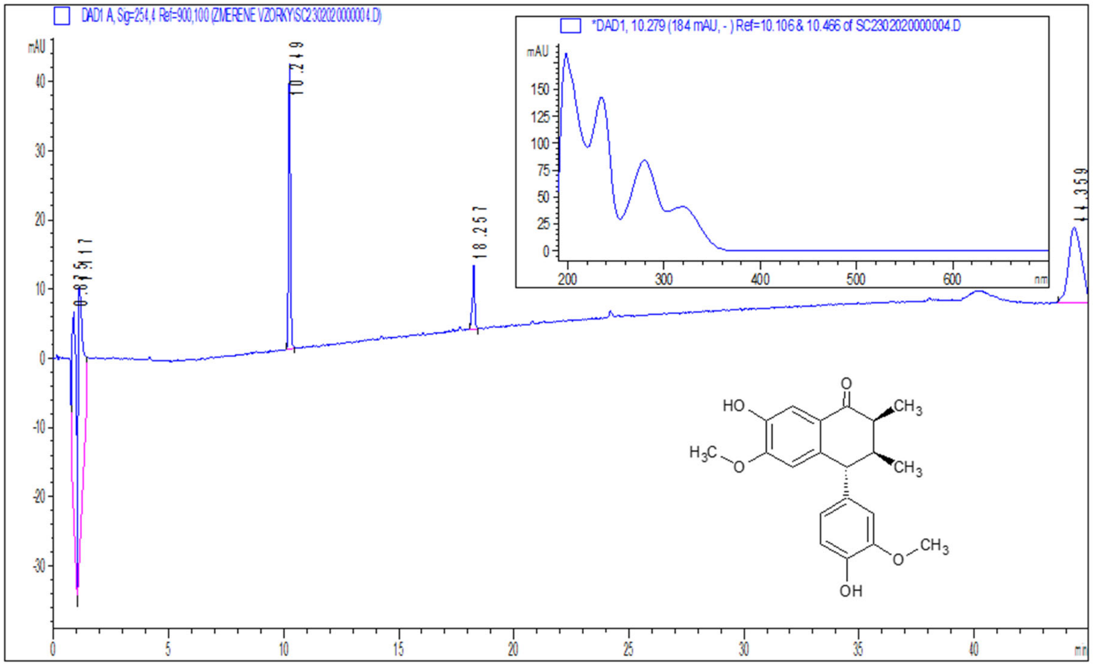

Figure S71. HPLC-DAD chromatogram of arisantetralone A

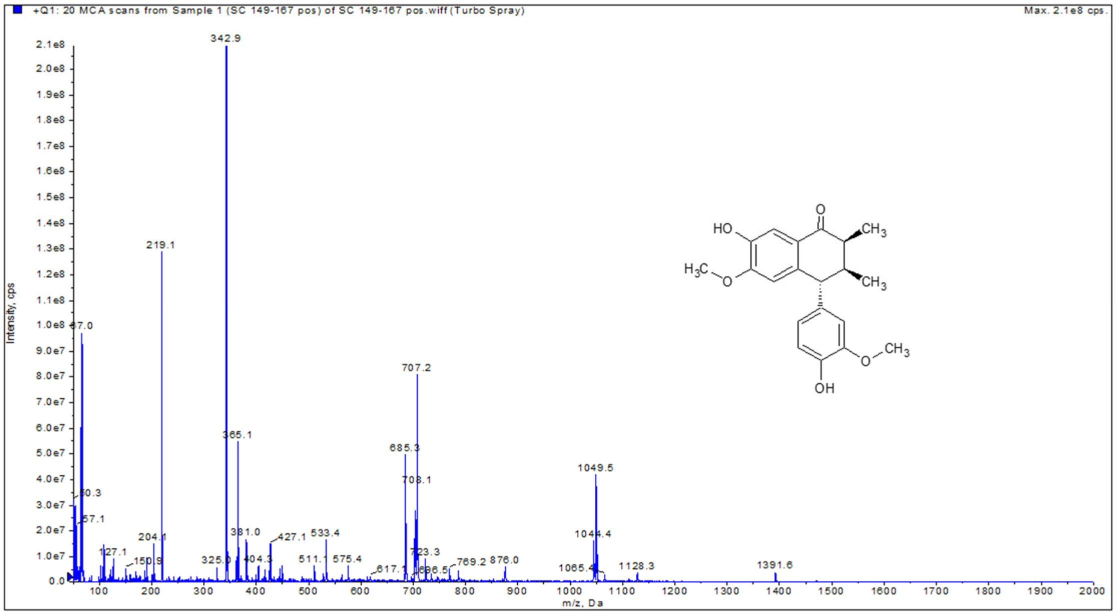

Figure S72. LR-MS spectrum of arisantetralone A

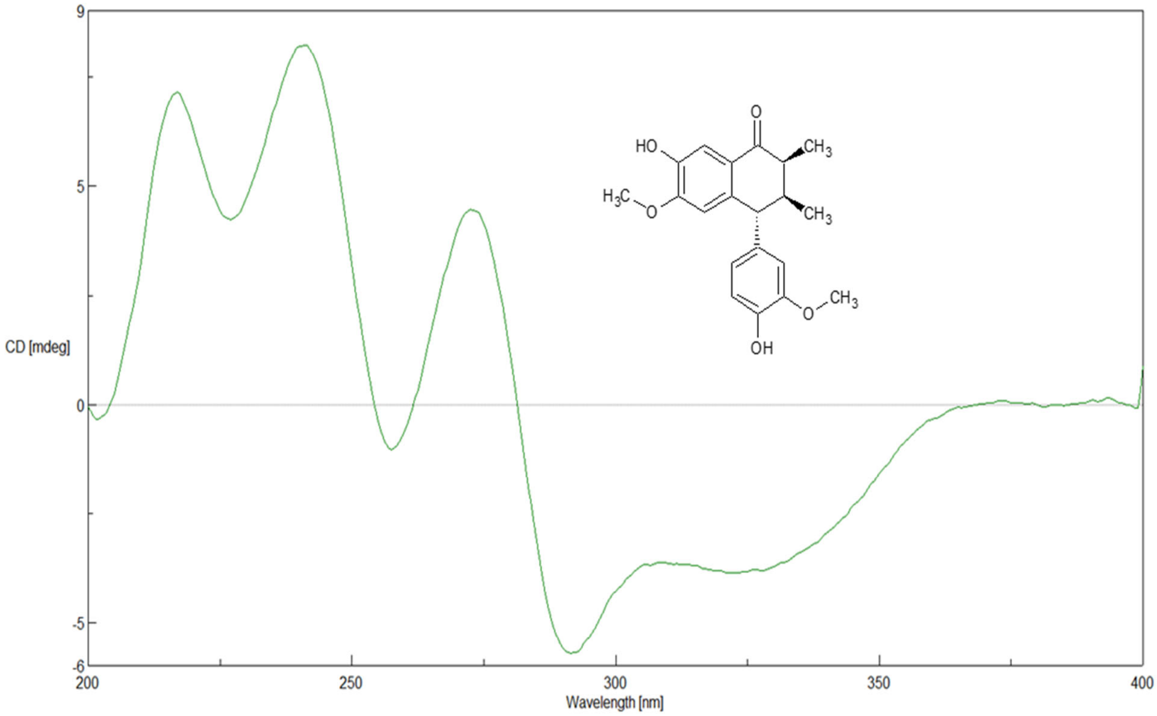

Figure S73. CD spectrum of arisantetralone A in methanol (c=1 mg/mL)

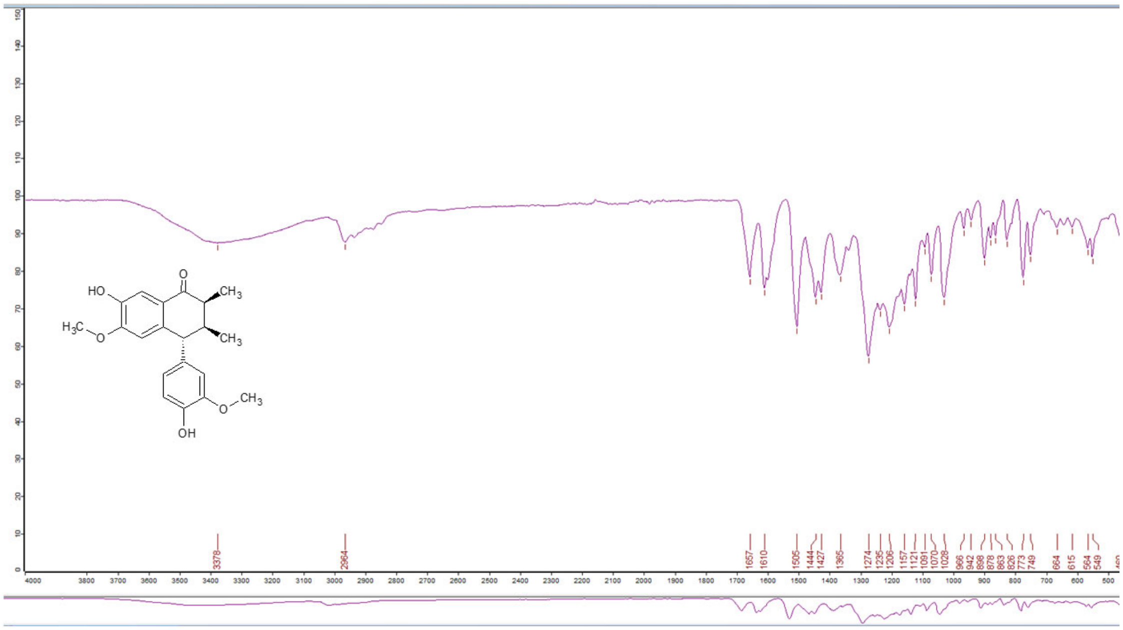

Figure S74. IR spectrum of arisantetralone A

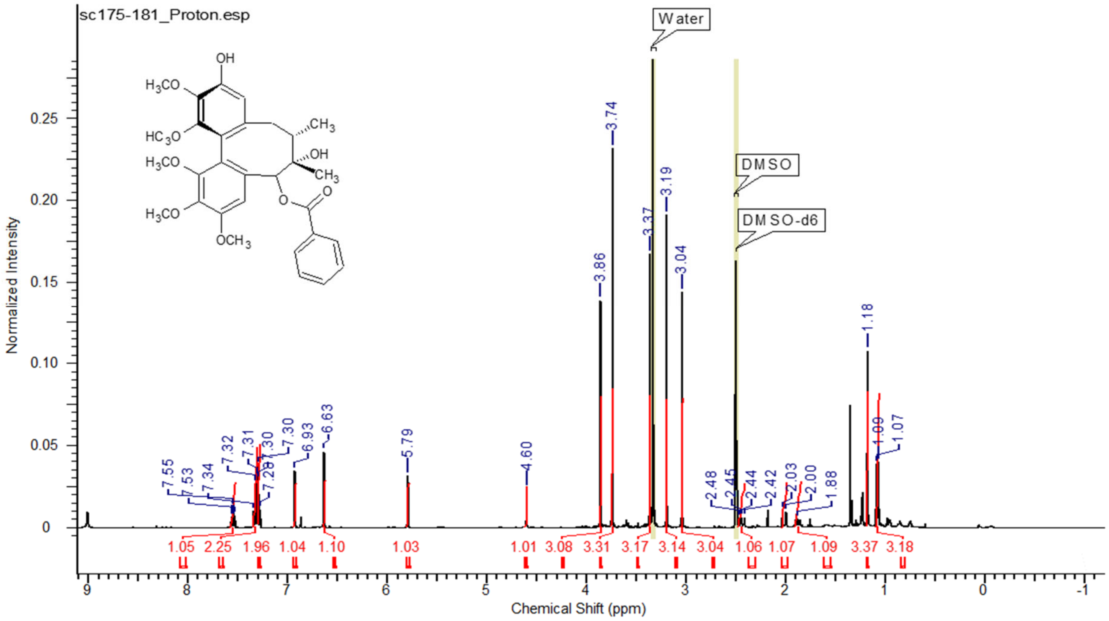

Figure S75. ^{1}H NMR spectrum of (−)-schisantherin E in DMSO-d_6

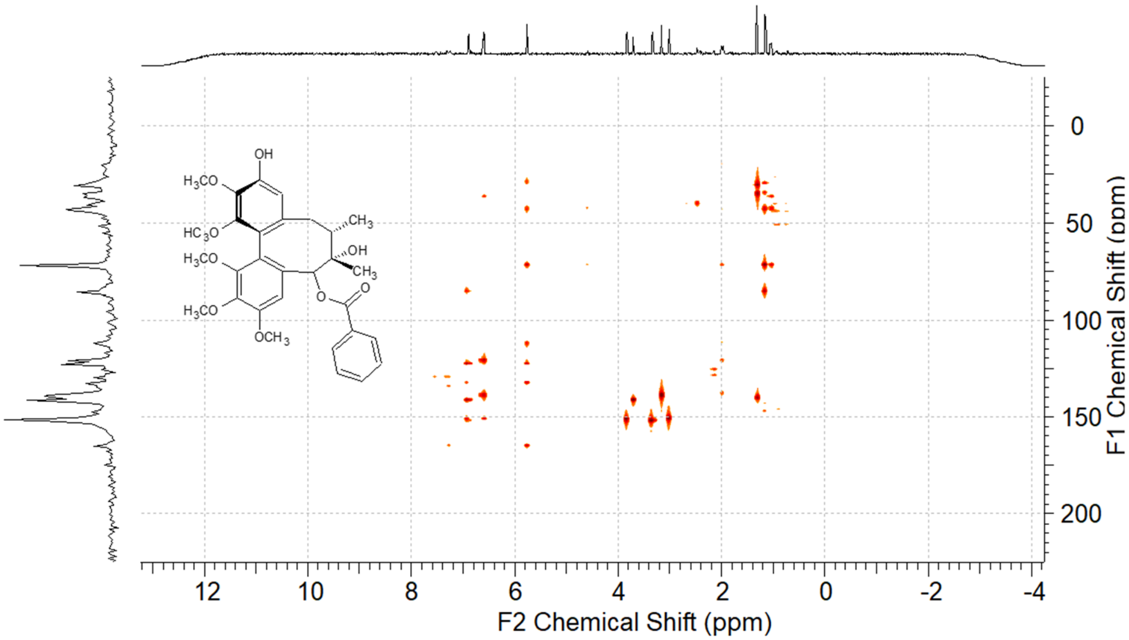

Figure S76. HMBC NMR spectrum of (−)-schisantherin E in DMSO-d_6

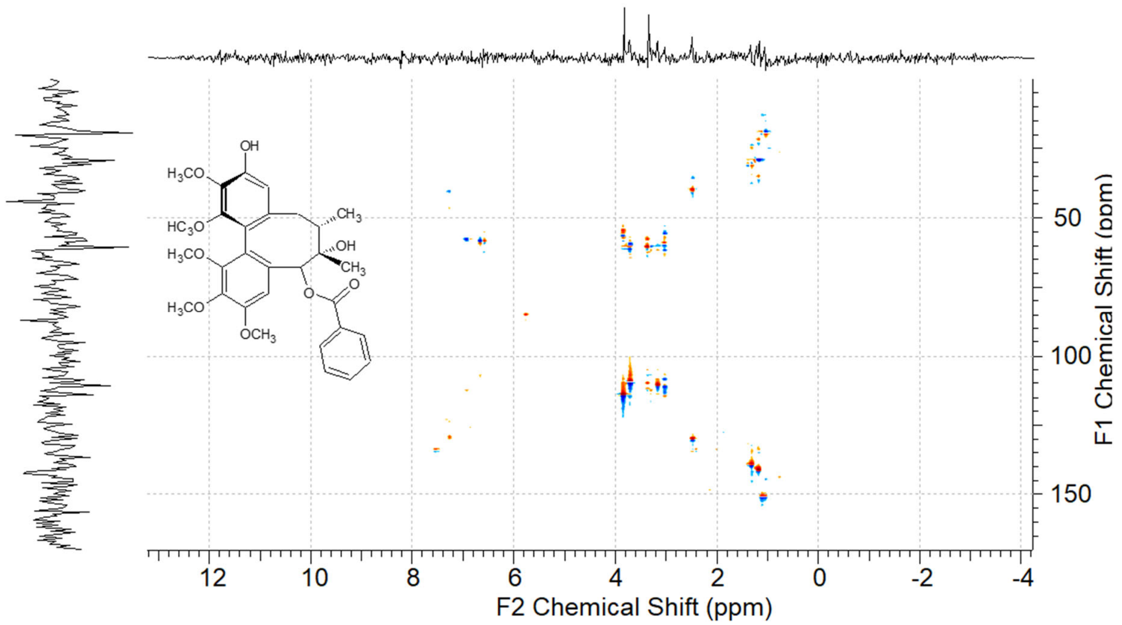

Figure S77. HSQC NMR spectrum of (−)-schisantherin E in DMSO-d_6

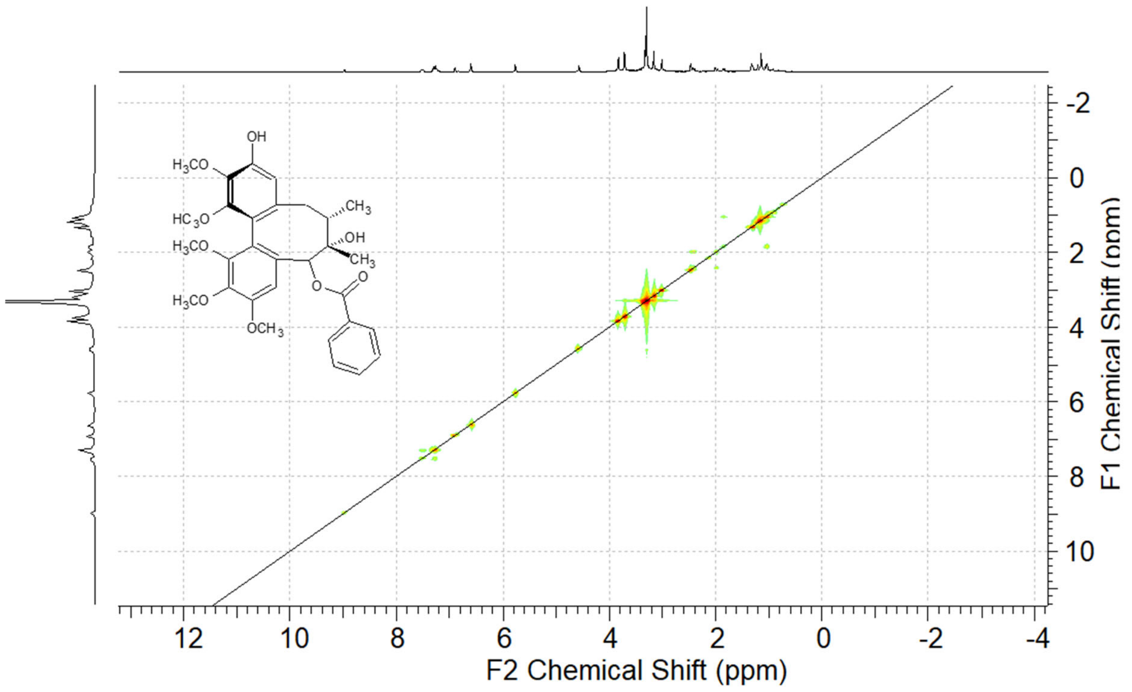

Figure S78. COSY NMR spectrum of (−)-schisantherin E in DMSO-d_6

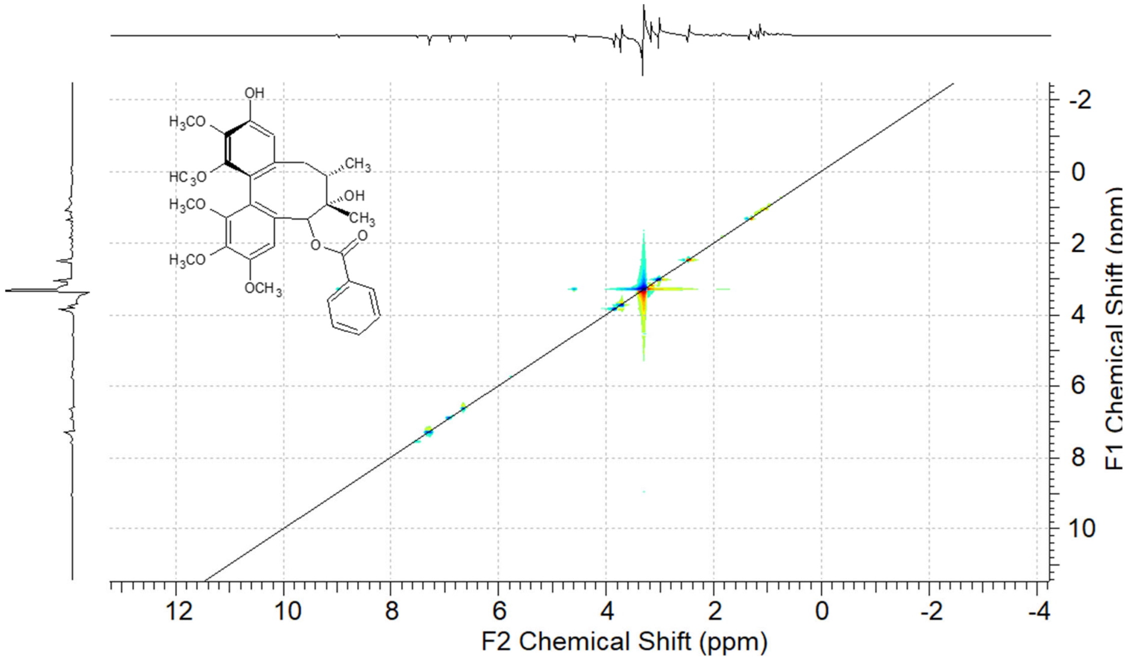

Figure S79. NOESY NMR spectrum of (−)-schisantherin E in DMSO-d_6

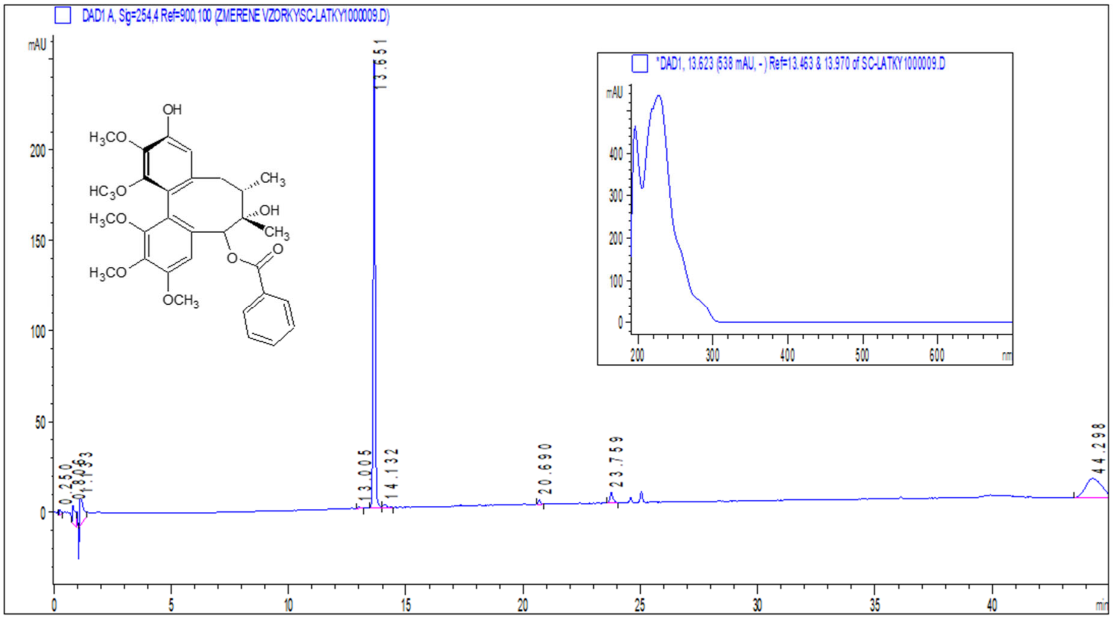

Figure S80. HPLC-DAD chromatogram of (−)-schisantherin E

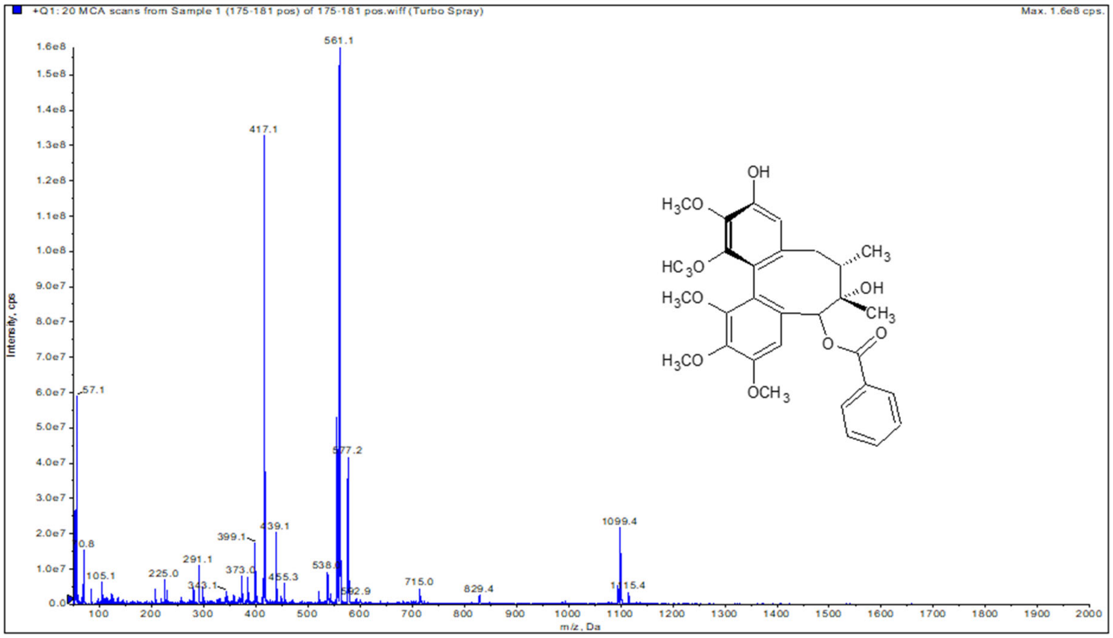

Figure S81. LR-MS spectrum of (−)-schisantherin E

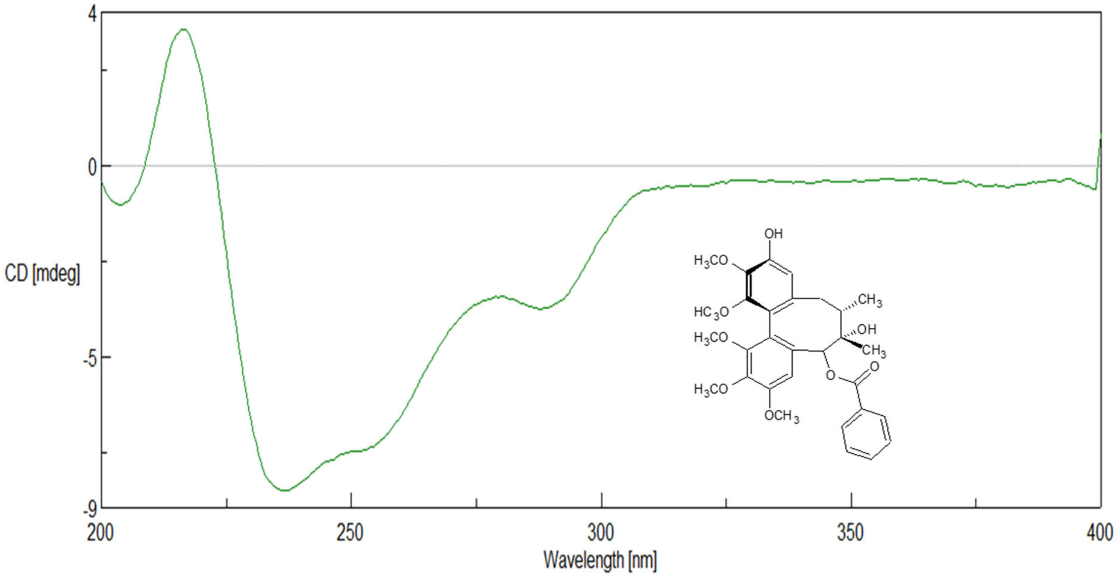

Figure S82. CD spectrum of (−)-schisantherin E in methanol (c=1 mg/mL)

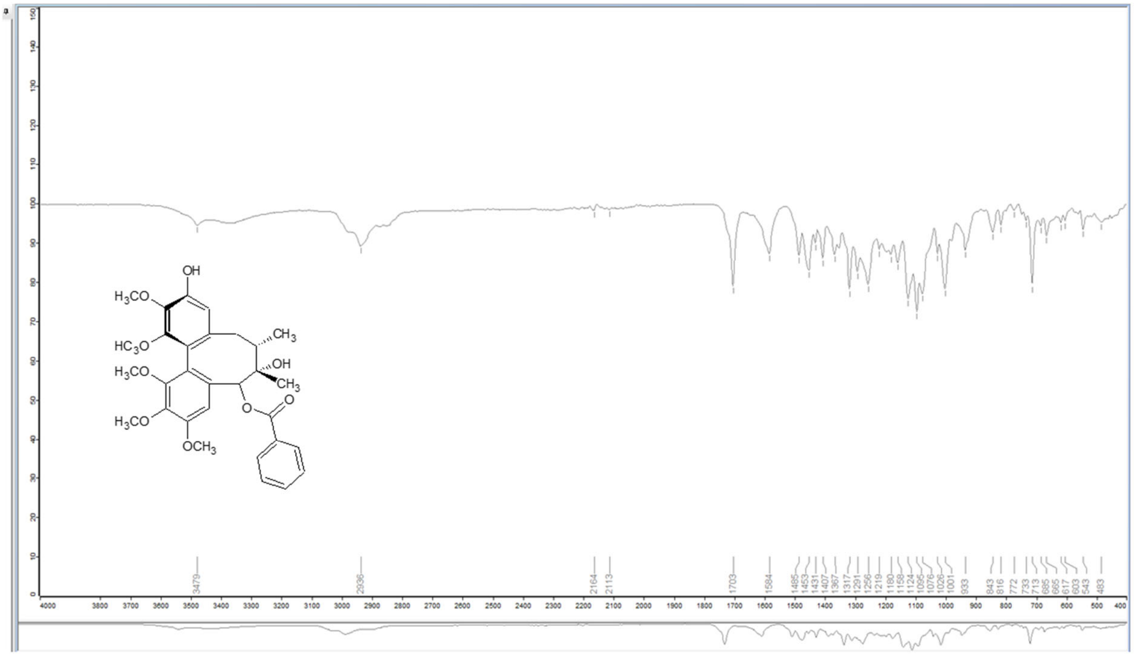

Figure S83. IR spectrum of (−)-schisantherin E

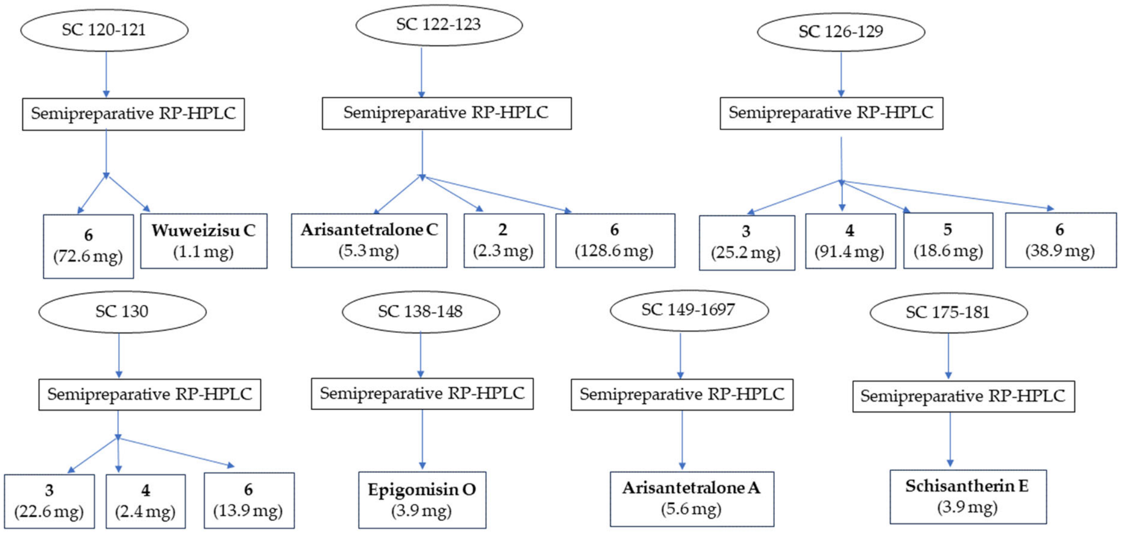

Figure S84. Schema of isolation and total yields of compounds **2–6**, (−)-wuweizisu C, arisantetralone A, arisantetralone C, epigomisin O, and (−)-schisantherin E

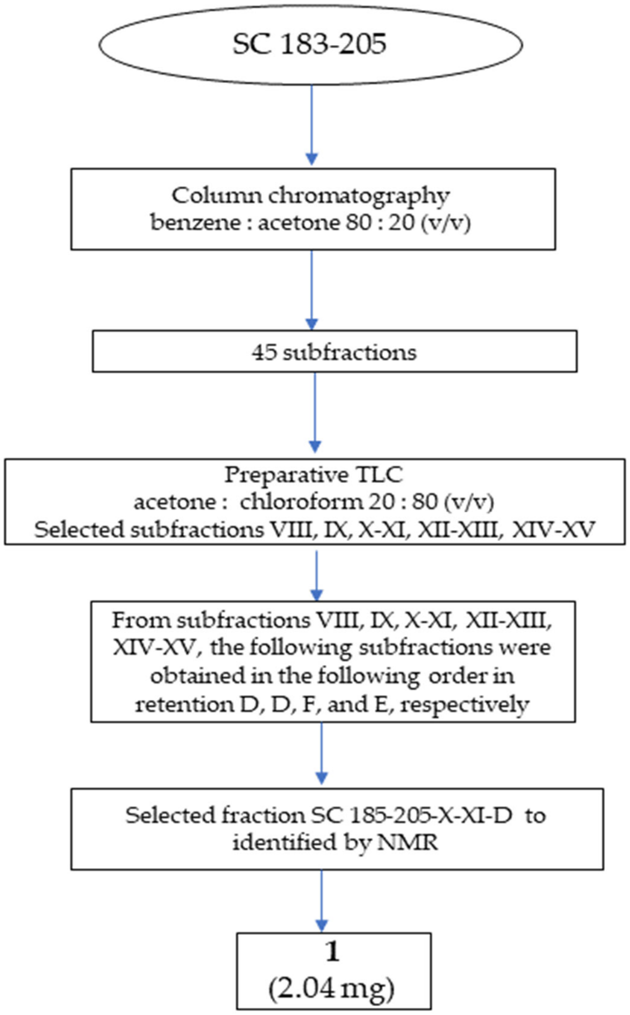

Figure S85. Schema of isolation and yield of compound **1**

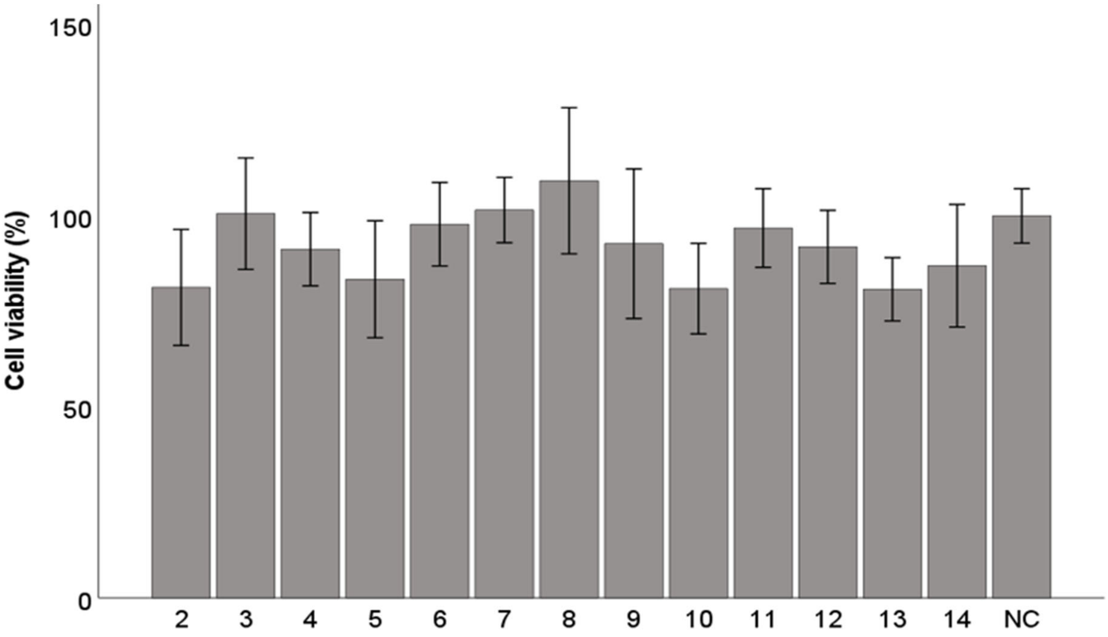

Figure S86. Antiproliferative activity of lignans **2–14**, expressed as % of cell viability. The concentration of tested lignans **2–14** was 10 μM. DMSO was used as the solvent and was added as the negative control (NC). The results are expressed as the mean ± SEM for three independent experiments measured in triplicate and are statistically compared to NC using Kruskal-Wallis test.

Table S1**.** Chromatographic conditions of separation of compounds **2**, **6**, and arisantetralone C from fraction SC 122-123

Stationary phase: Ascentis RP-Amide 25 cm × 10 mm; 5 μm (Supelco, USA)
Detection: DAD detector λ = 220; 240; 260; 280 nm
Flow:5 mL/min
Injection:40 μl
Temperature:40 °C
HPLC Dionex Ultimate 3000 (Thermo Fischer Scientific, USA)

**Time (min)**	**MeOH (%)**	**ACN (%)**	**0.2 % HCOOH (%)**
0	0	47	53
29.00	0	85	15
29.10	100	0	0
35.00	100	0	0
35.10	0	100	0
40.00	0	100	0
40.10	0	47	53
45.00	0	47	53

Table S2. Chromatographic conditions of separation of compounds **3**, **4**, **5** and **6** from fraction SC 130

Stationary phase: Ascentis RP-Amide 25 cm × 10 mm; 5 μm (Supelco, USA)
Detection: DAD detector λ = 240; 260; 280 nm
Flow:5 mL/min
Injection: 15 μL
Temperature:40 °C
YL9100 (Young-Lin Instruments, South Korea)

Time (min)	MeOH (%)	ACN (%)	0.2 % HCOOH (%)
0	0	50	50
30.00	0	80	20
30.01	0	100	0
35.00	0	100	0
35.01	100	0	0
40.00	100	0	0
40.01	0	50	50
45.00	0	50	50

Table S3. Chromatographic conditions of purification of compound **3** from fraction SC 130

Stationary phase: Ascentis RP-Amide 25 cm × 10 mm; 5 μm (Supelco, USA)
Detection: DAD detector λ = 240; 260; 280 nm
Flow:5 mL/min
Injection: 30 μL
Temperature: 40 °C
HPLC Dionex Ultimate 3000 (Thermo Fischer Scientific, USA)

Time (min)	MeOH (%)	ACN (%)	0.2 % HCOOH (%)
0	0	55	45
18.00	0	64	36
18.10	100	0	0
23.00	100	0	0
23.10	0	55	45
28.00	0	55	45

Table S4. Chromatographic conditions of the first purification of compounds **4** and **5** from fraction SC 130

Stationary phase: Ascentis RP-Amide 25 cm × 10 mm; 5 μm (Supelco, USA)
Detection: DAD detector λ = 240; 260; 280 nm
Flow: 5 mL/min
Injection: 25 μL
Temperature: 40 °C
HPLC Dionex Ultimate 3000 (Thermo Fischer Scientific, USA)

**Time (min)**	**MeOH (%)**	**ACN (%)**	**0.2 % HCOOH (%)**
0	0	55	45
18.00	0	64	36
18.10	100	0	0
23.00	100	0	0
23.10	0	55	45
28.00	0	55	45

Table S5. Chromatographic conditions of the second purification of compounds **4** and **5** from fraction SC 130

Stationary phase: Ascentis C18 25 cm × 10 mm, 5 μm (Supelco, USA)
Detection: DAD detector λ = 220, 240; 260; 280 nm
Flow: 5 mL/min
Injection: 10 μL
Temperature: 40 °C
HPLC Dionex Ultimate 3000 (Thermo Fischer Scientific, USA)

**Time (min)**	**MeOH (%)**	**ACN (%)**	**0.2 % HCOOH (%)**
0	60	0	40
18.00	73	0	27
18.10	0	100	0
23.00	0	100	0
23.10	60	0	40
28.00	60	0	40

Table S6. Chromatographic conditions of separation of compound **6** and (−)-wuweizisu C from fraction SC 120-121

Stationary phase: Ascentis RP-Amide 25 cm × 10 mm; 5 μm (Supelco, USA)
Detection: DAD detector λ = 220; 240; 260; 280 nm
Flow: 5 mL/min
Injection: 10 μL
Temperature:40 °C
system YL9100 (Young-Lin Instruments, South Korea)

Time (min)	MeOH (%)	ACN (%)	0.2 % HCOOH (%)
0	0	30	70
30.00	0	80	20
30.01	0	100	0
35.00	0	100	0
35.01	100	0	0
40.00	100	0	0
40.01	0	30	70
45.00	0	30	70

Table S7. Chromatographic conditions of separation of epigomisin O from fraction SC 138-148

Stationary phase: Ascentis RP-Amide 25 cm × 10 mm; 5 μm (Supelco, USA)
Detection: DAD detector λ = 220; 240; 260; 280 nm
Flow: 5 mL/min
Injection: 25 μL
Temperature:40 °C
HPLC Dionex Ultimate 3000 (Thermo Fischer Scientific, USA)

Time (min)	MeOH (%)	ACN (%)	0.2 % HCOOH (%)
0	60	0	40
20.00	79	0	21
20.10	0	100	0
28.00	0	100	0
28.10	100	0	0
36.00	100	0	0
36.10	60	0	40
45.00	60	0	40

Table S8. Chromatographic conditions of separation of arisantetralone A from fraction SC 149-167

Stationary phase: Ascentis RP-Amide 25 cm × 10 mm; 5 μm (Supelco, USA)
Detection: DAD detector λ = 240; 260; 280 nm
Flow: 5 mL/min
Injection:10 μL
Temperature: 40 °C
system YL9100 (Young-Lin Instruments, South Korea)

Time (min)	MeOH (%)	ACN (%)	0.2 % HCOOH (%)
0	0	40	60
20.00	0	60	40
20.01	0	100	0
25.00	0	100	0

25.01	100	0	0
30.00	100	0	0
30.01	0	40	60
45.00	0	40	60

Table S9**.** Chromatographic conditions of separation of (−)- schisantherin E from fraction SC 175-181

Stationary phase: Ascentis RP-Amide 25 cm × 10 mm; 5 μm (Supelco, USA)
Detection: DAD detector λ = 240; 260; 280 nm
Flow: 5 mL/min
Injection: 20 μL
Temperature: 40 °C
system YL9100 (Young-Lin Instruments, South Korea)

Time (min)	MeOH (%)	ACN (%)	0.2 % HCOOH (%)
0	0	30	70
30.00	0	90	10
30.01	0	100	0
35.00	0	100	0
35.01	100	0	0
40.00	100	0	0
40.01	0	30	70
45.00	0	30	70

Table S10**.** Chromatographic conditions of separation of compounds **3**, **4**, **5**, and **6** from fraction SC 126-129

Stationary phase: Ascentis RP-Amide 25 cm × 10 mm; 5 μm (Supelco, USA)
Detection: DAD detector λ = 220, 240; 260; 280 nm
Flow: 5 mL/min
Injection: 15 μL
Temperature: 40 °C
HPLC Dionex Ultimate 3000 (Thermo Fischer Scientific, USA)

Time (min)	MeOH (%)	ACN (%)	0.2 % HCOOH (%)
0	0	40	60
29.00	0	81	19
29.10	100	0	0
35.00	100	0	0
35.10	0	100	0
40.00	0	100	0
40.10	0	40	60
45.00	0	40	60

Table S11**.** Chromatographic conditions of purification of compounds **4** and **5** from fraction SC 126-129

Stationary phase: Ascentis C18 25 cm × 10 mm, 5 μm (Supelco, USA)<br>Detection: DAD detector λ = 220, 240; 260; 280 nm<br>Flow: 5 mL/min<br>Injection: 15 μL<br>Temperature: 40 °C<br>HPLC Dionex Ultimate 3000 (Thermo Fischer Scientific, USA)			
**Time (min)**	**MeOH (%)**	**ACN (%)**	**0.2 % HCOOH (%)**
0	70	0	30
19.00	82.9	0	17.1
19.10	0	100	0
24.00	0	100	0
24.10	70	0	30
29.00	70	0	30
